# Morphogenetic defects underlie Superior Coloboma, a newly identified closure disorder of the dorsal eye

**DOI:** 10.1371/journal.pgen.1007246

**Published:** 2018-03-09

**Authors:** Jennifer C. Hocking, Jakub K. Famulski, Kevin H. Yoon, Sonya A. Widen, Cassidy S. Bernstein, Sophie Koch, Omri Weiss, FORGE Canada Consortium, Seema Agarwala, Adi Inbal, Ordan J. Lehmann, Andrew J. Waskiewicz

**Affiliations:** 1 Division of Anatomy, Department of Surgery, University of Alberta, Edmonton, Canada; 2 Women & Children’s Health Research Institute, University of Alberta, Edmonton, Canada; 3 Department of Biological Sciences, University of Alberta, Edmonton, Canada; 4 Department of Medical Genetics, University of Alberta, Edmonton, Canada; 5 Department of Biology, University of Kentucky, Lexington, Unites States of America; 6 Department of Molecular Biosciences, University of Texas at Austin,Unites States of America; 7 Department of Medical Neurobiology, Institute for Medical Research–Israel-Canada, The Hebrew University-Hadassah Medical School, Jerusalem, Israel; 8 Institute for Cell and Molecular Biology, University of Texas at Austin, Austin, Unites States of America; 9 Institute for Neuroscience, University of Texas at Austin, Austin, Unites States of America; 10 Department of Ophthalmology, University of Alberta, Edmonton, Canada; 11 Neuroscience and Mental Health Research Institute, University of Alberta, Edmonton, Canada; Vanderbilt University Medical Center, UNITED STATES

## Abstract

The eye primordium arises as a lateral outgrowth of the forebrain, with a transient fissure on the inferior side of the optic cup providing an entry point for developing blood vessels. Incomplete closure of the inferior ocular fissure results in coloboma, a disease characterized by gaps in the inferior eye and recognized as a significant cause of pediatric blindness. Here, we identify eight patients with defects in tissues of the superior eye, a congenital disorder that we term *superior coloboma*. The embryonic origin of superior coloboma could not be explained by conventional models of eye development, leading us to reanalyze morphogenesis of the dorsal eye. Our studies revealed the presence of the *superior ocular sulcus* (SOS), a transient division of the dorsal eye conserved across fish, chick, and mouse. Exome sequencing of superior coloboma patients identified rare variants in a Bone Morphogenetic Protein (Bmp) receptor (*BMPR1A*) and T-box transcription factor (*TBX2*). Consistent with this, we find sulcus closure defects in zebrafish lacking Bmp signaling or Tbx2b. In addition, loss of dorsal ocular Bmp is rescued by concomitant suppression of the ventral-specific Hedgehog pathway, arguing that sulcus closure is dependent on dorsal-ventral eye patterning cues. The superior ocular sulcus acts as a conduit for blood vessels, with altered sulcus closure resulting in inappropriate connections between the hyaloid and superficial vascular systems. Together, our findings explain the existence of superior coloboma, a congenital ocular anomaly resulting from aberrant morphogenesis of a developmental structure.

## Author summary

Ocular coloboma is a disease characterized by gaps in the lower portion of the eye and can affect the iris, lens, or retina, and cause loss of vision. Coloboma arises from incomplete closure of a transient fissure on the underside of the developing eye. Therefore, our identification of patients with similar tissue defects, but restricted to the superior half of eye, was surprising. Here, we describe an ocular developmental structure, the *superior ocular sulcus*, as a potential origin for the congenital disorder *superior coloboma*. Formation and closure of the sulcus are directed by dorsal-ventral eye patterning, and altered patterning interferes with the role of the sulcus as a pathway for blood vessel growth onto the eye.

## Introduction

Aberrant ocular morphogenesis during embryonic development frequently results in reduced visual acuity or blindness. Morphological development of the eye begins with evagination of retinal precursors from the forebrain to produce bilateral optic vesicles and subsequent invagination of the associated ectoderm to create the lens [1,2]. Each optic vesicle reorganizes into a bilayered optic cup, with the distal (lens-facing) layer forming the presumptive neural retina and the proximal layer forming the retinal pigmented epithelium (RPE). To provide an entry point for vasculature and an exit pathway for axons of the optic nerve, a transient inferior (choroid) fissure forms along the ventral/inferior side of the optic cup and stalk. In cases where the inferior fissure fails to close, gaps remain within tissues of the eye (iris, retina, choroid and/or occasionally lens) [3,4]. This congenital anomaly, referred to as ocular coloboma, is estimated to occur in 1 out of 4–5,000 live births and cause 3–11% of pediatric blindness [4,5]. Ocular coloboma has a complex causality encompassing mutations in over 20 genes [5,6]. Although both clinically and genetically heterogeneous, coloboma predominantly affects the inferior aspect of the eye.

The posterior segment of the developing eye receives two vascular supplies [7]. The transient hyaloid vasculature is a plexus between the retina and lens, and is connected to the hyaloid artery, which enters the eye via the inferior fissure. A second circulatory system, the choroidal vasculature, grows over the surface of the optic cup to nourish the RPE and the light-sensing photoreceptor cells in the outer retina. Although development of the choroidal vessels is poorly understood, zebrafish studies demonstrated that the complex choroidal vascular plexus is preceded by a simple set of pioneer vessels [8,9]. To form this so-called superficial vascular system (distinct from the superficial retinal vessels and also known as the ciliary vasculature), three radial vessels grow over the optic cup and anastomose to create an annular vessel encircling the lens. The highly stereotypical formation of the superficial vessels suggests precise developmental regulation, but the mechanisms that guide their growth are currently unknown.

In the context of studying a large cohort of patients with ocular coloboma, we identified five local patients with a novel ocular anomaly characterized by gaps in tissues of the superior eye. Although it is logical that such an anomaly represents another fissure disorder, common models of vertebrate eye development do not feature a division in the embryonic dorsal/superior eye. However, a careful examination of zebrafish, chick, and mouse eye development did reveal a transient groove, or sulcus, bisecting the dorsal optic cup. Moreover, we utilized patient exome sequencing and zebrafish models to define the importance of dorsal-ventral patterning in morphogenesis of this ocular sulcus. Functionally, the superior ocular sulcus serves as a conduit for the advancing first vessel of the superficial vasculature, and we note profound errors in vascular growth and connectivity in embryos with abnormal sulci.

## Results

### Identification of patients with superior coloboma

**Fig 1:**
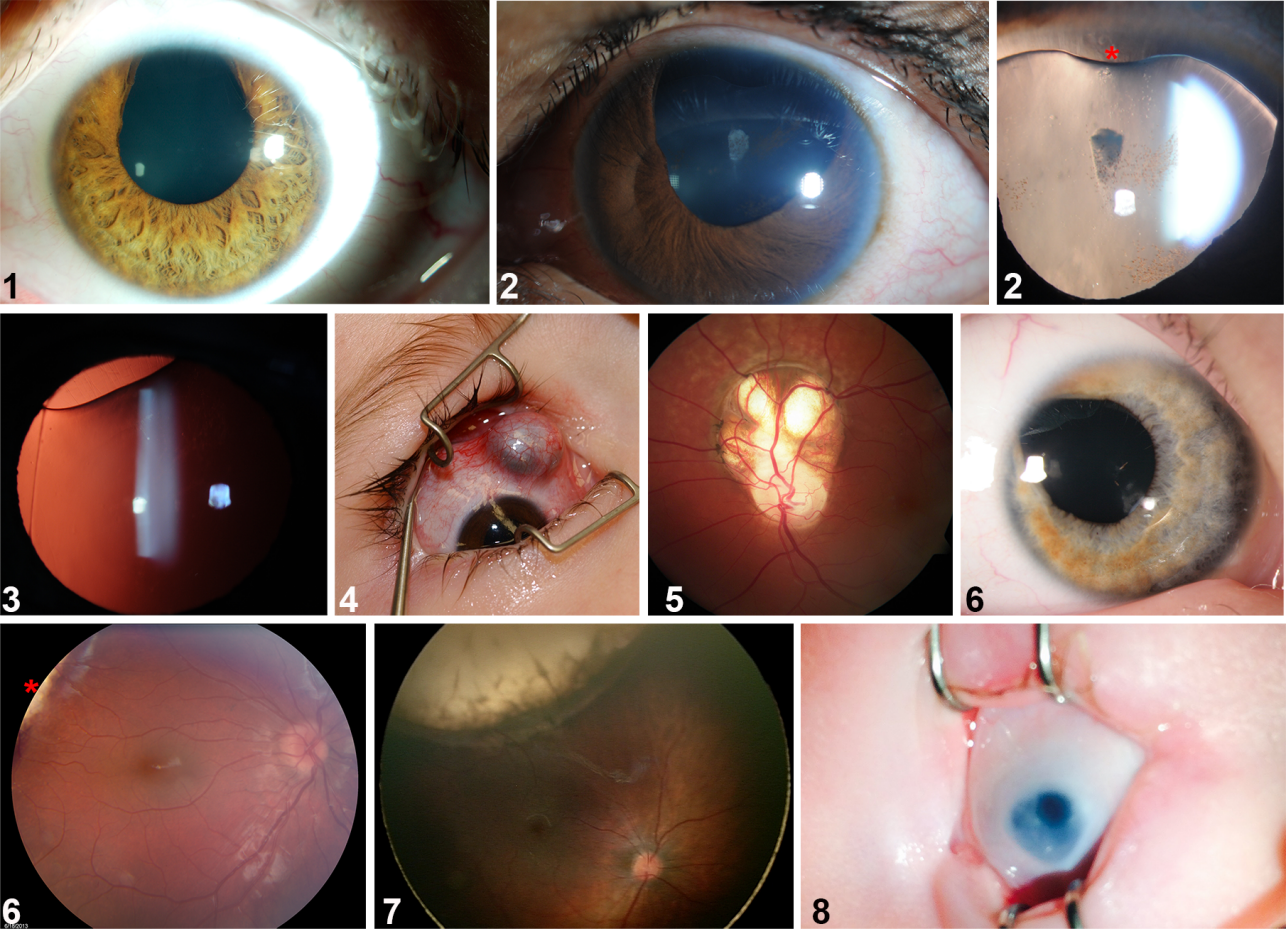
Superior coloboma. Montage from patients with superior coloboma (numbers represent patients described in S1 Table). #1: unilateral superior iris coloboma. #2: first panel, asymmetrically-sized iris defects with bilateral pupil involvement, left eye shown; second panel, superior lenticular coloboma (asterisk) associated with a lens zonule defect. #3: lenticular coloboma, lens edge visible with retro-illumination. #4: superior scleral defect with uveal (choroid) protrusion. #5: superior retino-choroidal coloboma extending from optic disc in patient with Dandy-Walker Syndrome. #6: first panel, iris coloboma; second panel, edge of retino-chorodial coloboma (asterisk). #7: extensive retino-choroidal coloboma. #8: intra-operative photograph of a superior iris coloboma in a microphthalmic eye.

Over a six-year period (2007–2012), we identified five local patients with superior ocular defects affecting the iris, lens, retina, optic nerve and/or sclera (Fig 1 and S1 Table); notably, these were unassociated with a family history of such anomalies. On the basis of apparent similarity to coloboma (gaps in inferior/ventral ocular tissue), yet inverse orientation, we propose the term *superior coloboma* to describe this disorder. The first patient, with tuberous sclerosis attributable to a rare *TSC2* (c.C5026T; p.R1676W) mutation, exhibited a prominent unilateral iris coloboma situated at 12 o'clock. Bilateral disease was present in a single patient (#2), and involved both iris and lens (Fig 1, images 2 and 3). Two of the five patients were diagnosed in infancy, and for one (#4), examination under anesthesia was required to fully characterize pathology. As is evident from Fig 1, the diversity of tissue involvement in superior colobomata recapitulates that present in inferior colobomata. We subsequently received, from pediatric ophthalmologists at US and UK tertiary referral centers, clinical data on three further patients with superior colobomata. These cases extended the range of associated phenotypes to include additional structural ocular malformations (microphthalmia, or small eye; #8). All eight patients in our cohort had profoundly reduced visual acuity, precluding normal stereopsis.

### Exome sequencing of superior coloboma patients

To identify candidate genetic variants carried by superior coloboma patients, exome sequencing was performed on the initial five probands (S2 Table). Identified variants were prioritized by comparison to SNP databases (frequency <1%), in silico prediction algorithms (Mutation Taster >0.95) and expression within the developing eye or previously identified connections to coloboma (see methods). We focused our efforts on understanding genetic alterations in the single patient with bilateral superior coloboma (#2, Table 1). In particular, we noticed that patient #2 carries compound heterozygous variants in the Retinoic Acid (RA) synthesis gene *CYP1B1* [10](S1A Fig) as well as a rare (dbSNP: 1 in 60,706; NHLBI and 1000 Genomes: 0 in 14,000) missense variant in Bone Morphogenetic Protein Receptor 1A (*BMPR1A*, S1B and S1C Fig). As RA and BMPs are morphogens with essential roles in eye development, including regulation of inferior fissure closure [11–17], we hypothesized that the identified mutations contributed to the patient’s ocular disorders. In order to examine how disruption of eye patterning genes could lead to superior coloboma, we next turned to animal models and conducted an in depth analysis of dorsal eye morphogenesis.

**Table 1:** Select genetic variants identified in superior coloboma patient #2.

Gene	Type	Variant	Mutation Taster
			
ACVRL1	nonsynonymous SNV	NM_001077401:c.C1445T:p.A482V	0.999706
ASPM	nonsynonymous SNV	NM_018136:c.C4213T:p.R1405C	0.999919
SYNE1	nonsynonymous SNV	NM_033071:c.G12229C:p.D4077H	0.990532
TLR10	nonsynonymous SNV	NM_001017388:c.T1255C:p.W419R	0.989287
CD36	stopgain SNV	NM_001127444:c.T1079G:p.L360X	1
COL10A1	nonsynonymous SNV	NM_000493:c.T23G:p.L8W	0.998959
CTSC	nonsynonymous SNV	NM_001814:c.A1088C:p.E363A	0.999985
CYP1A1	nonsynonymous SNV	NM_000499:c.C712T:p.P238S	0.999742
CYP1A1	nonsynonymous SNV	NM_000499:c.T857C:p.I286T	0.99848
CYP1B1	nonsynonymous SNV	NM_000104:c.G1103A:p.R368H	0.970216
DHRS9	nonsynonymous SNV	NM_001142271:c.G856C:p.D286H	0.99818
DHX38	nonsynonymous SNV	NM_014003:c.A2947G:p.I983V	0.999192
DOCK5	nonsynonymous SNV	NM_024940:c.G2698A:p.E900K	0.993012
DSCAM	nonsynonymous SNV	NM_001389:c.G701A:p.R234H	0.979737
EAF1	nonsynonymous SNV	NM_033083:c.G619A:p.D207N	0.976956
FURIN	nonsynonymous SNV	NM_002569:c.G1343A:p.R448Q	0.997805
HHIP	nonsynonymous SNV	NM_022475:c.C1762T:p.P588S	0.999972
IFT57	nonsynonymous SNV	NM_018010:c.A1232G:p.N411S	0.991636
LAMA4	nonsynonymous SNV	NM_001105206:c.G3239A:p.R1080Q	0.997947
LAMA5	nonsynonymous SNV	NM_005560:c.G10411A:p.G3471S	0.999959
LAMC2	nonsynonymous SNV	NM_005562:c.C2080T:p.R694C	0.994374
LRP2	nonsynonymous SNV	NM_004525:c.G13803A:p.M4601I	0.999811
MCM5	nonsynonymous SNV	NM_006739:c.G375C:p.Q125H	0.999992
MKS1	stopgain SNV	NM_001165927:c.C478T:p.R160X	1
MMP2	nonsynonymous SNV	NM_001127891:c.C1481T:p.S494L	0.994768
MMP9	nonsynonymous SNV	NM_004994:c.A344G:p.K115R	0.985048
NCOR1	nonsynonymous SNV	NM_001190440:c.G6956A:p.R2319Q	0.966004
NEUROD1	nonsynonymous SNV	NM_002500:c.C590A:p.P197H	0.999982
PCDH15	stopgain SNV	NM_001142767:c.T1283G:p.L428X	1
PLXNA3	nonsynonymous SNV	NM_017514:c.A3440G:p.K1147R	0.996782
SIPA1L1	nonsynonymous SNV	NM_015556:c.C3056T:p.T1019M	0.99995
SLIT2	nonsynonymous SNV	NM_004787:c.G4333C:p.D1445H	0.993067
SOD2	nonsynonymous SNV	NM_000636:c.G198C:p.E66D	0.999998
SYNE1	nonsynonymous SNV	NM_033071:c.G12229C:p.D4077H	0.990532
TLR10	nonsynonymous SNV	NM_001017388:c.T1255C:p.W419R	0.989287

### Vertebrate studies of dorsal ocular morphogenesis

**Fig 2:**
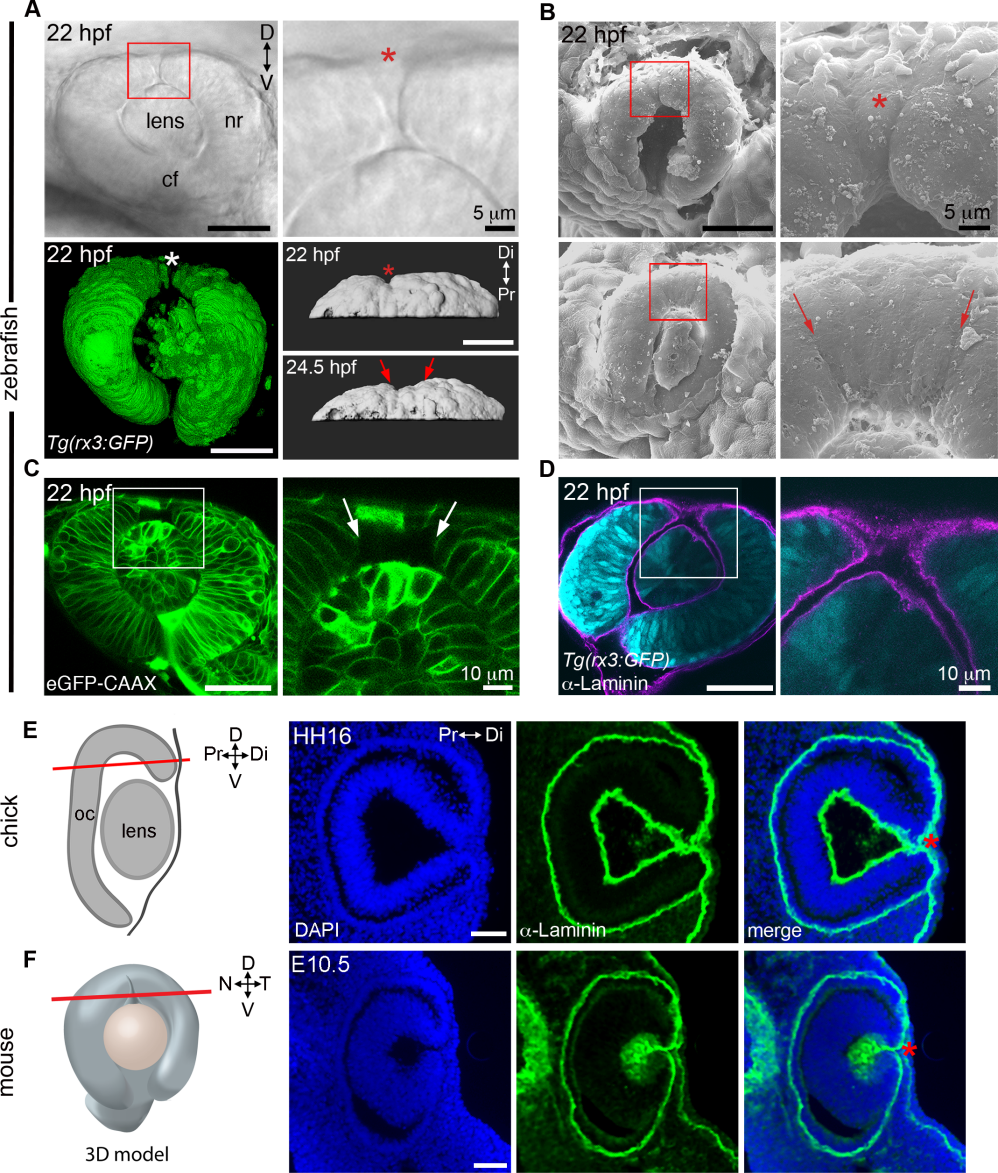
The superior ocular sulcus in zebrafish, chick and mouse. (A) Zebrafish eyes displaying superior ocular sulci (SOS) marked by an asterisk or arrows. Top row: lateral view DIC image of the eye of a live embryo, photographed on a compound microscope. Enlarged view is shown in panel on right. Bottom row: Left, lateral view surface projection of the eye of a live *Tg(rx3*:*GFP)* embryo; Right, surface projection dorsal views of eyes from a *Tg(rx3*:*GFP)* embryo. (B) Scanning electron micrographs showing SOS at narrow (top row) and wide (bottom row) phases. Red boxes denote regions enlarged in panels on the right. (C) Single optical section, lateral view, through the eye of an embryo injected with *eGFP-CAAX* mRNA to label the cell membranes, with right panel showing enlarged view of boxed area. (D) Single optical section, lateral view, through eye of *Tg(rx3*:*GFP)* embryo (cyan) immunolabelled for Laminin to highlight the basal lamina (magenta). (E) Diagram showing chick eye with red line demonstrating the plane of section employed on the right. Representative horizontal section through the dorsal eye of a HH16 chick, stained with a Laminin antibody (green) and DAPI (blue). A dorsal, Laminin-lined space is evident in the distal portion of optic cup (asterisk). (F) Diagram showing 3D model of an embryonic eye with red line demonstrating plane of section for both mouse and chick sections. Right three panels are a representative horizontal section through the dorsal eye of an embryonic day 10.5 (E10.5) mouse, stained with a Laminin antibody (green) and DAPI (blue). A dorsal, Laminin-lined space is evident in the distal portion of optic cup (asterisk). Except where noted, scale bars are 50 μm. cf, choroid fissure; D-V, dorsal-ventral; HH, Hamburger Hamilton embryonic stage; hpf, hours post fertilization; N-T, nasal-temporal; nr, neural retina; Pr-Di, proximal-distal.

Inferior coloboma arises from failed closure of the choroid fissure located in the ventral eye. Given the comparable phenotype despite opposite orientation seen in superior coloboma patients, we hypothesized a similar etiology. Although the standard model of eye development describes an uninterrupted dorsal retina, two older studies of fish eye development identified a groove present in this space [18,19]. To determine if such a structure exists broadly across vertebrates and whether it is a Laminin-lined space, we chose to revisit the study of dorsal eye morphogenesis in fish, chick and mouse. Using multiple microscopy methods, we identified a transient groove/sulcus in the dorsal zebrafish eye [dorsal in fish and superior in human are equivalent; for consistency with superior coloboma, we describe this structure as the *superior ocular sulcus* (SOS)] (Fig 2A–2D). The sulcus is visible by stereoscope but more obvious in compound or confocal observations of live embryos (Fig 2A), and most easily discernible from 21–25 hpf. When imaged under an electron microscope, the SOS can be seen to transect the distal portion of the dorsal retina (Fig 2B), while single confocal optical slices reveal the SOS as a distinct space (Fig 2C) lined by basal lamina (Fig 2D).

To ascertain whether a similar structure exists in chick, we examined tissue sections immunostained for Laminin and counterstained with DAPI. At stage HH16, we observed the presence of a Laminin-lined division in the distal portion of the chick dorsal optic cup (n = 6/8 eyes; Fig 2E, S2 Fig). For evidence of a comparable structure in mammals, we next examined mice and found a Laminin-lined separation across the inferior portion of the dorsal optic cup at embryonic day 10.5 (Fig 2F). A collaborator also shared older SEM studies of newt (*Taricha tarosa*) development, which similarly demonstrate the presence of a division across the dorsal embryonic eye (S3 Fig, personal communication, A. Jacobson). Thus, we present clear evidence for the existence of an evolutionarily conserved, Laminin-lined sulcus in the dorsal optic cup of multiple vertebrate species.

The inferior fissure temporarily bisects the ventral retina prior to closing through progressive fusion of the nasal and temporal margins of the ventral optic cup [3]. The SOS similarly extends across the dorsal zebrafish retina (Fig 2A–2D) to partially separate the nasal and temporal retinal lobes, and is also present only transiently. To determine the mechanism of SOS closure, we followed ocular morphogenesis over time. The SOS arises soon after optic cup formation (19–20 hpf) as a distinct and narrow structure (S1 Video and Fig 2A and 2B). Notably, formation of the sulcus occurs at a time when the developing retinal pigmented epithelium is spreading around the optic cup, but is not associated with significant cell movement or apoptosis in the forming dorsal retina (S2 Video). Unexpectedly, the edges of the narrow SOS do not migrate toward one another and fuse, but instead the SOS transitions at 22–24 hpf to a shallow and wide trough and gradually disappears after 26 hpf (S3–S5 Videos and S4 Fig). Both phases are visible in representative scanning electron microscopy images (Fig 2B). As we observed the transition from narrow to wide, and never detected an epithelial fusion event, it is logical to propose that the sulcus closes via cell rearrangement or shape modification, mechanisms distinct from the epithelial fusion that occurs within the choroid fissure.

### *CYP1B1* and the superior ocular sulcus

*CYP1B1* mutations cause ocular malformation and are a major cause of congenital and adult glaucoma [20]. Patient #2 carries one of the known disease-causing alleles (R368H) while the second allele is a truncation (A287Pfs6), and so both alleles are expected to be pathogenic. Retinoic acid can be synthesized through both the Cyp1b1 and Aldh pathways [10,21], and mRNA encoding both types of RA synthesis enzyme is expressed in the dorsal zebrafish eye (S5A Fig)[12,22]. In order to test whether Cyp1b1 is necessary for SOS closure, we used TALEN mutagenesis to create zebrafish carrying a 13 bp frameshift deletion within the P450 domain, resulting in an early stop codon and a truncated protein. Surprisingly, the zebrafish *cyp1b1* mutants did not display defects in sulcus closure, even when the Aldh pathway was additionally inhibited (S5B Fig)[23]. Given the lack of a phenotype with reduced RA signaling, we next investigated the *BMPR1A* variant and Bmp-dependent regulation of SOS closure.

### Bmp signaling regulates closure of the superior ocular sulcus

**Fig 3:**
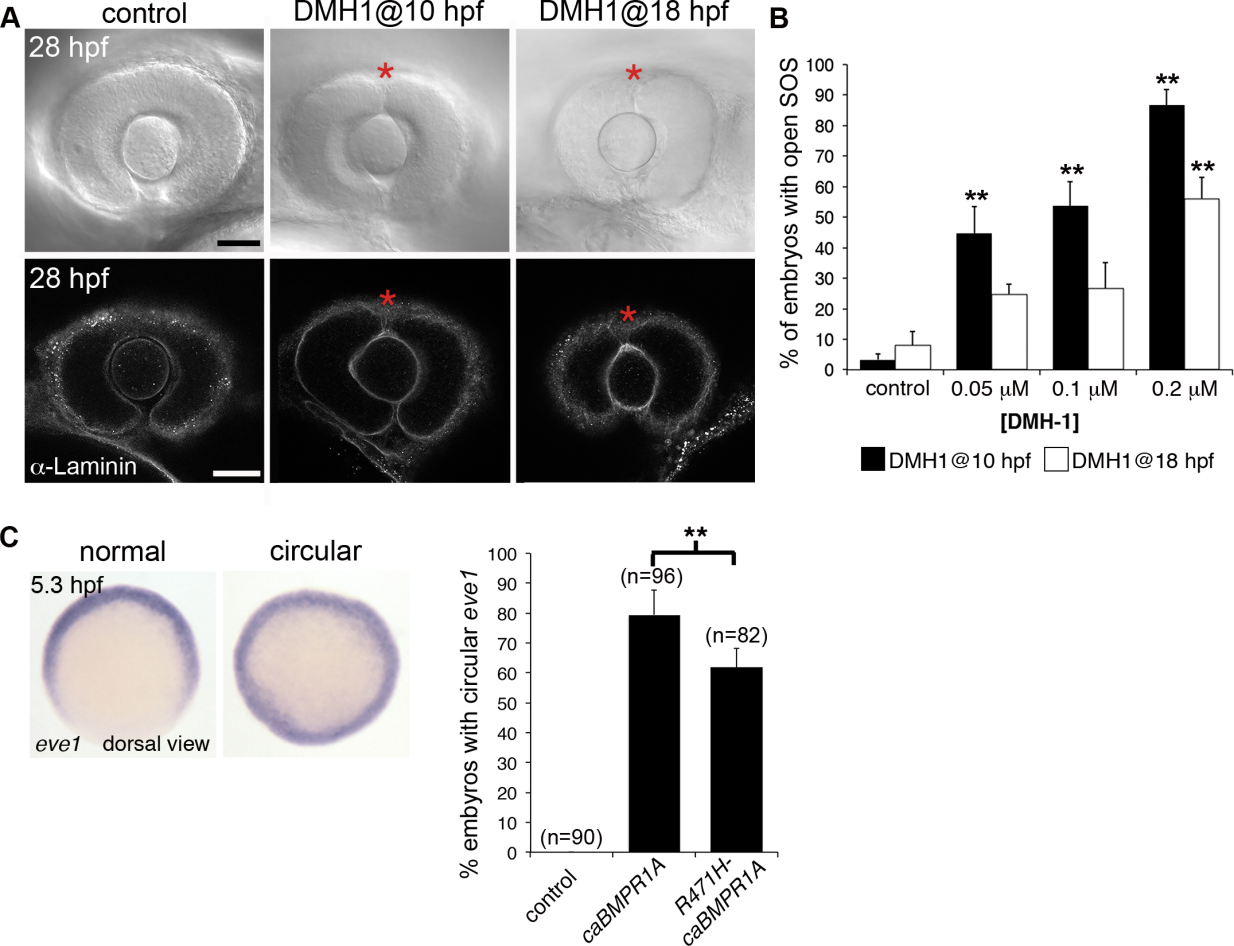
The role of BMPR1 signaling in closure of the superior ocular sulcus. (A-B) Effect of Bmpr1 antagonist DMH1 on SOS closure. Lateral view DIC images of eyes from live embryos (first row) and single optical slices of eyes processed for anti-Laminin immunofluorescence (second row) following exposure to control media or 0.02 μM DMH1, starting at either 10 or 18 hpf (A). SOS is marked by red asterisk. Quantification of delayed sulcus closure in DMH1-treated embryos (B). N = 3 experiments, n = 89 or 90 embryos for each condition. Data are means ± SEM. Statistics is a one-way ANOVA for each time series with Tukey's post-hoc test: **P<0.01. (C) Injection of *caBMPR1A* mRNA into one-cell stage zebrafish embryos caused expansion of *eve1* gene expression into a circular ring in whole embryos at 50% epiboly (5.3 hpf). Significantly fewer embryos exhibited circular *eve1* expression when injected with *R471H-caBMPR1A*. N = 3 experiments. Data are means ± SEM. Statistics is a two-tailed *t* test: *P<0.05. Scale bars are 50 μm.

Bmp ligands (Gdf6/Bmp13 and Bmp 2, 4, and 7) pattern the eye at the time of SOS closure [11–14,24,25] and the identified *BMPR1A* patient variant alters a highly conserved residue in the kinase domain (p.Arg471His, S1 Fig); therefore, we tested whether reduced Bmp receptor activity affects closure of the SOS. The small molecule DMH1 is an inhibitor of type IA BMP receptors, with robust and specific activity in zebrafish [26,27]. Embryos were treated with DMH1 either just after gastrulation or just prior to optic cup invagination (10 and 18 hpf, respectively) and evaluated for SOS presence at 28 hpf, a time point when the sulcus is no longer visible in wildtype embryos. Exposure to DMH1 prevented SOS closure in a dose-dependent manner (Fig 3A and 3B), establishing that Bmp signaling regulates sulcus morphogenesis.

We next used a zebrafish overexpression assay to evaluate whether the patient variant disrupts BMPR1A function. As injection of one-cell stage embryos with wildtype human *BMPR1A* mRNA failed to elicit alterations to dorsal-ventral axis specification, we used site-directed mutagenesis to introduce a Q233D mutation previously shown to render BMPR1A constitutively active [28]. Injection of mRNA encoding the constitutively active BMPR1A receptor (caBMPR1A) efficiently induced ventralization of whole zebrafish embryos, while *caBMPR1A* carrying the patient variant (*R471H-caBMPR1A*) showed mildly reduced activity (Fig 3C and S6 Fig). The patient variant therefore does not completely inactivate the protein, but this assay does suggest that it could be a hypomorphic allele and may have been one of multiple factors contributing to the development of superior coloboma. Overall, our data support a role for Bmp signaling in regulating SOS closure.

**Fig 4:**
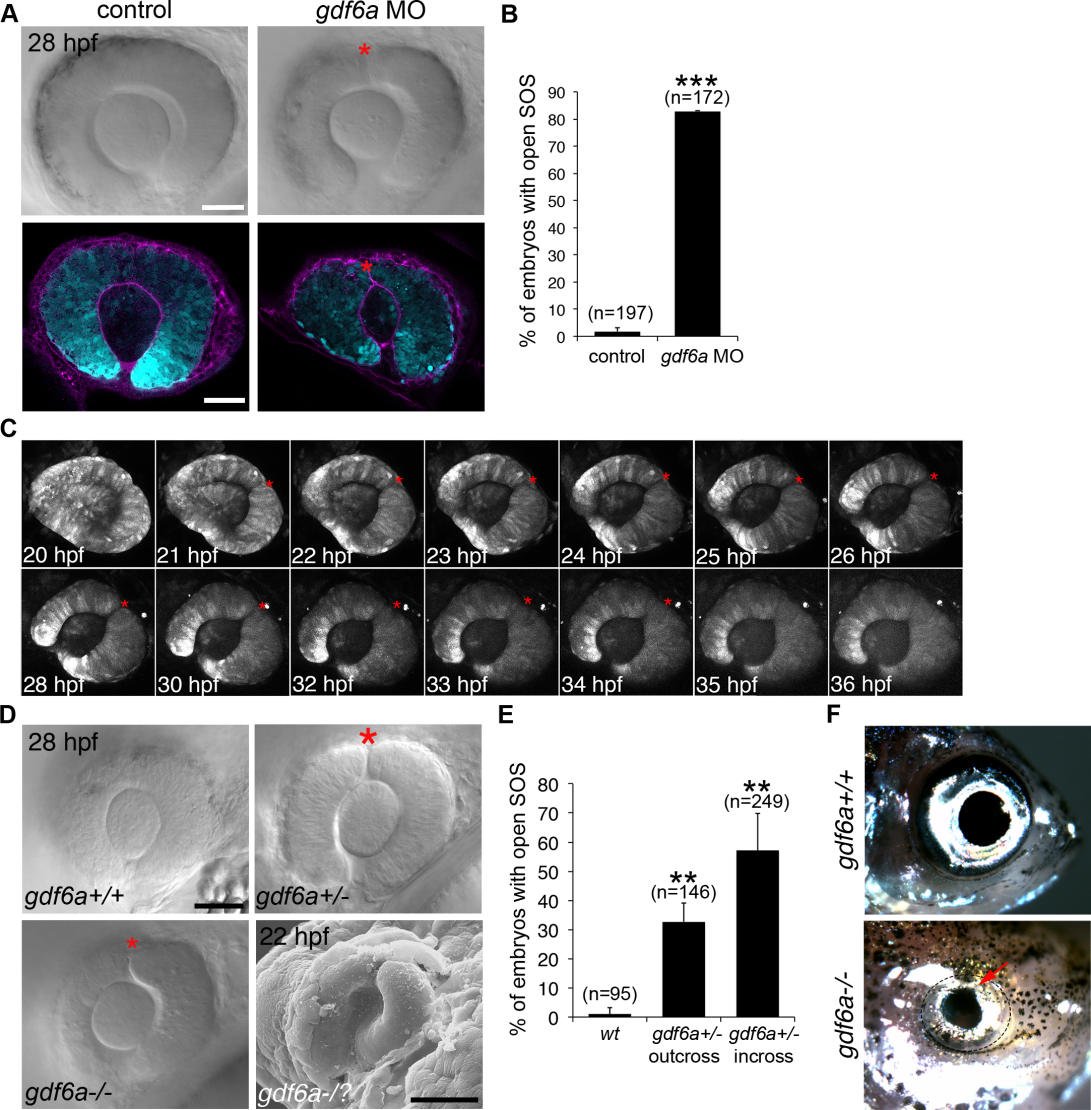
The role of Gdf6a signaling in superior ocular sulcus morphogenesis. (A) Delayed SOS closure caused by Gdf6a knockdown. *Tg(rx3*:*GFP)* zebrafish eyes (cyan) from uninjected and Gdf6a morpholino-injected embryos shown as DIC images of live embryos and single optical slices following anti-Laminin antibody staining (magenta). SOS marked by red asterisk. (B) Quantification of embryos with delayed sulcus closure, as assessed at 28 hpf. (C) Time series of maximum projection confocal images of a *Tg(rx3*:*GFP)* embryo injected with *gdf6a* morpholino. (D) DIC images of wildtype, *gdf6a*^+/-^ and *gdf6a*^-/-^ eyes (SOS marked by red asterisk). Bottom right panel shows SEM image of a Gdf6a-deficient eye with a pronounced sulcus. (E) Quantification of *gdf6a*^*-/-*^ mutants (or siblings) with delayed SOS closure. (F) Adult wildtype zebrafish (top panel) showing normal eye morphology and a *gdf6a*^*-/-*^ zebrafish (bottom panel) with superior coloboma (red arrow). N = 3 experiments for graphs in B and E. n = number of embryos. Data are means ± SEM. Statistics in B is a two-tailed *t* test, and in E is one-way ANOVA with Tukey’s test: **P<0.01, *** *P*<0.001. Scale bars are 50 μm.

Within the zebrafish eye, Bmpr1a mediates signaling from the Gdf6a (Growth Differentiation Factor 6a, Bmp13) ligand [29] and absence of Gdf6a results in almost complete loss of dorsal (superior) ocular genes, expansion of ventral (inferior) genes, and a small eye phenotype [12,13,30]. Knockdown of Gdf6a signaling in wildtype embryos by injection of antisense morpholino oligonucleotides caused a highly penetrant SOS closure defect, very similar to that seen with DMH1 exposure (Fig 4A–4C). Recapitulation of the persistent sulcus phenotype in both homozygous [12,13] and a subset of heterozygous *gdf6a* embryos (Fig 4D and 4E) shows that SOS closure is sensitive to the precise level of Bmp signaling. A lack of Gdf6a also affected formation of the SOS, as seen by the deeper sulcus in a representative SEM image (Fig 4D, bottom right panel) and in animations showing the surface morphology of the dorsal eye in 22 hpf wildtype (S6 Video), *gdf6a* heterozygous (S7 Video) and *gdf6a* homozygous (S8 Video) embryos. While the sulcus eventually closes in most Gdf6a-deficient embryos, two adult *gdf6a*^*-/-*^ fish displayed superior colobomata (Fig 4F), demonstrating that an early closure defect can lead to the disease phenotype.

**Fig 5:**
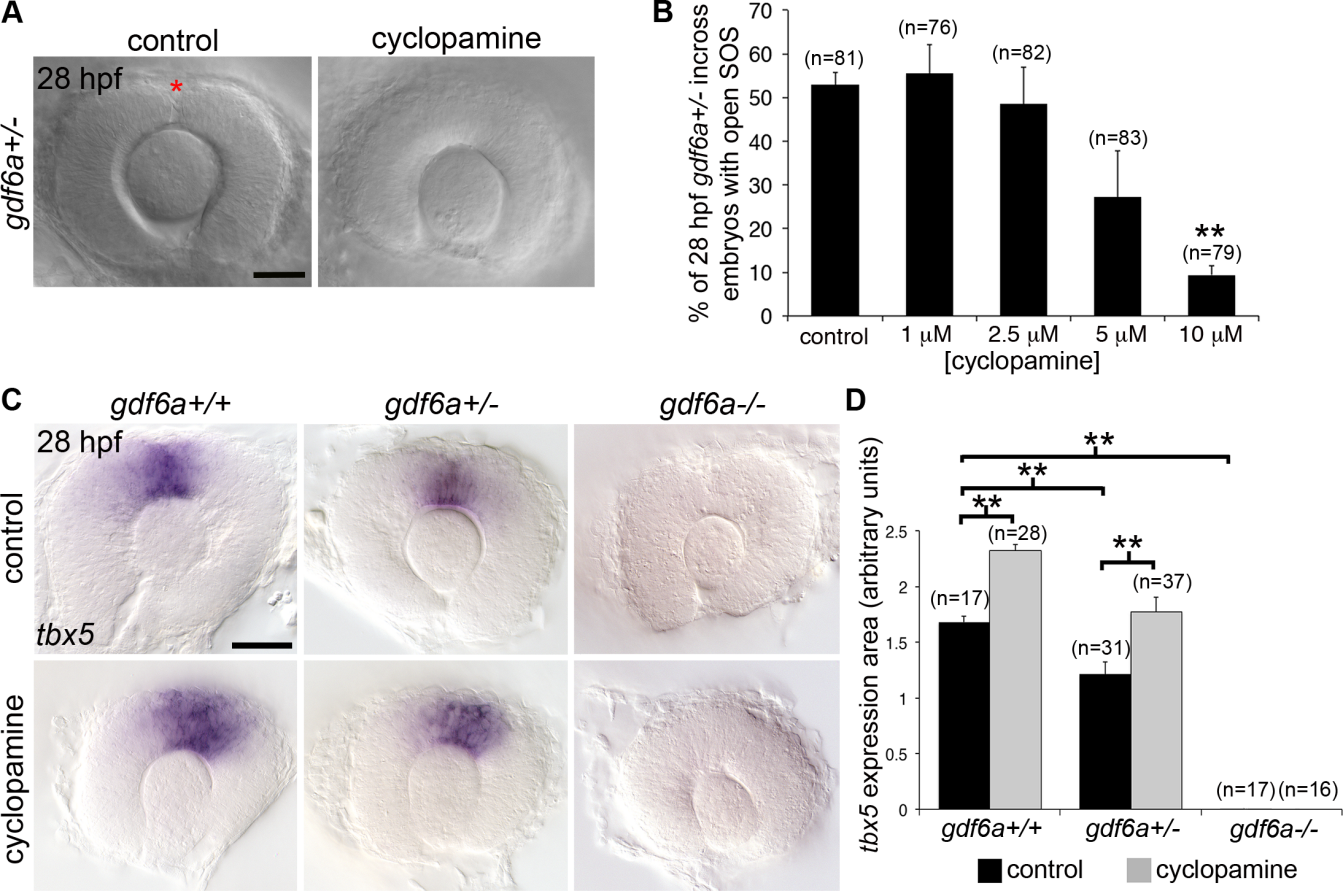
Inhibition of Hedgehog signaling rescues closure of the superior ocular sulcus in Gdf6a-deficient embryos. (A-B) Effect of Hedgehog inhibition (cyclopamine treatment) on SOS closure in Gdf6a-deficient embryos. DIC images of *gdf6a*^+/-^ eyes, treated with either control solution (left) or 10 μM cyclopamine (right) (A). SOS marked by red asterisk. Quantification of effect of cyclopamine treatment on SOS closure in *gdf6a*^+/-^ incross embryos (B). (C-D) Effect of cyclopamine on dorsal retinal patterning in Gdf6a-deficient embryos. *tbx5* RNA expression in eyes from 28 hpf *gdf6a*^*+/+*^, *gdf6a*^*+/-*^, and *gdf6a*^*-/-*^ embryos with or without cyclopamine treatment (C). Quantification of effect of cyclopamine treatment on area of *tbx5* expression (D). n = number of embryos, N = 4 (B) or 3 (D) experiments. Data are means ± SEM. Data in B and D are means ± SEM; Statistics in B is a one-way ANOVA with Tukey’s test, D is two-way ANOVA with Tukey’s test: **P<0.01. Scale bars are 50 μm.

There are diverse outputs of Gdf6a signaling, regulating cellular functions such as apoptosis, cell proliferation, and dorsal-ventral retinal patterning [12,13,17,31]. Because proliferative defects are visible after sulcus closure and apoptotic cells are not concentrated near the SOS [31], we reasoned that dorsal-ventral retinal patterning is the Gdf6a function most essential for SOS closure. During development, dorsal ocular Bmp signaling is balanced by midline Sonic Hedgehog (Shh) activity [16,32], and *gdf6a*^*-/-*^ mutants exhibit an expansion of the Shh downstream gene *vax2* into the dorsal retina [13]. We therefore tested whether increased Shh signaling in Bmp-deficient embryos underlies the persistent SOS phenotype. Indeed, treatment of *gdf6a*^*-/-*^ and *gdf6a*^*+/-*^ embryos with the Shh inhibitor cyclopamine significantly rescued the delayed closure phenotype (Fig 5A and 5B). Cyclopamine treatment also partially rescued patterning in the dorsal retina, as it restored the *tbx5* expression domain in *gdf6a* heterozygotes (Fig 5C and 5D). These data support the idea that SOS closure is dependent on proper pattern formation within the developing retina and that sulcus morphogenesis is regulated by a balance of ventral Shh and dorsal Bmp signaling pathways.

### Analysis of a second superior coloboma variant

**Fig 6:**
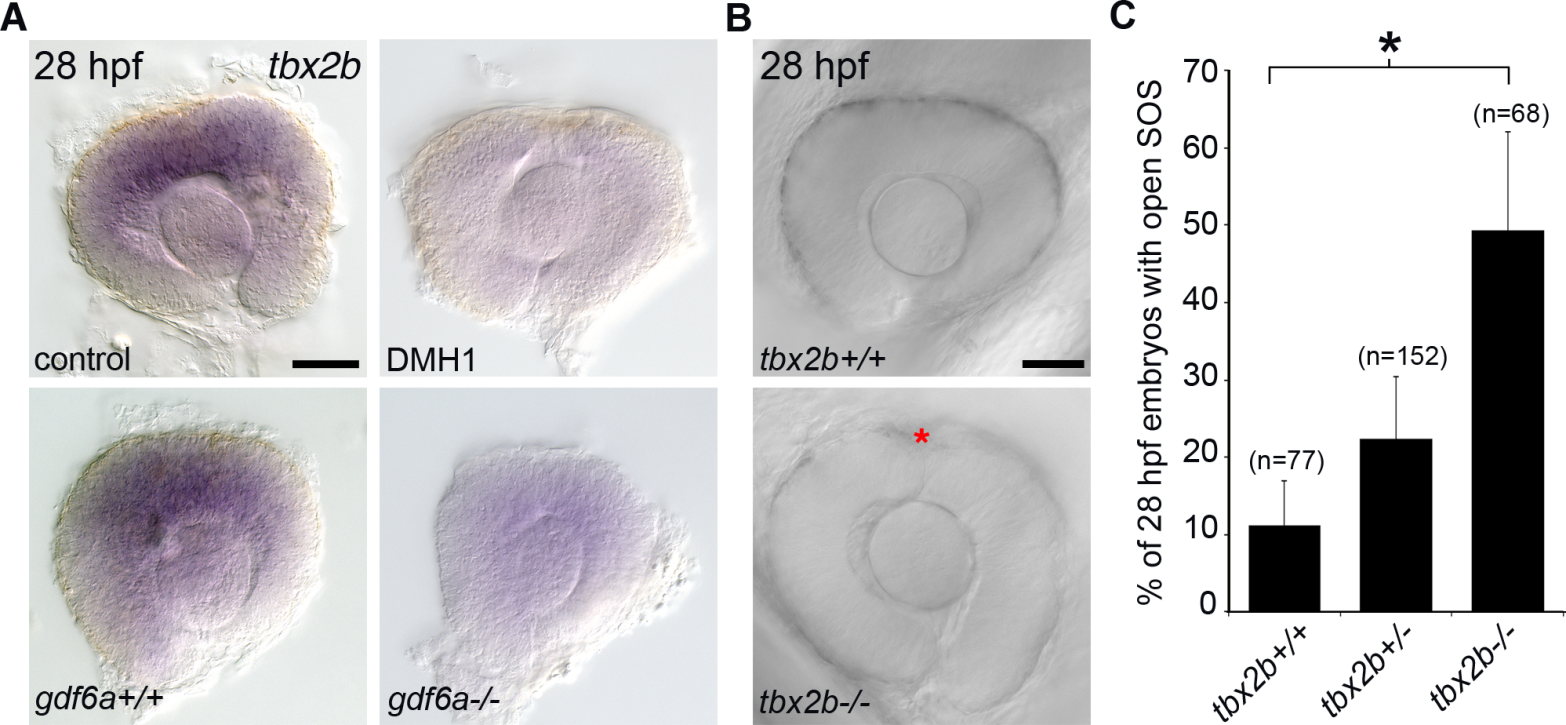
Analysis of Tbx2b and closure of the superior ocular sulcus. (A) Whole-mount in situ hybridization for zebrafish *tbx2b* in control and BMP-depleted embryos. Top panels are eyes dissected from control and DMH1-treated embryos; bottom panels are from *gdf6a*^*+/+*^, and *gdf6a*^*-/-*^ embryos. (B-C) Analysis of SOS closure in Tbx2b-depleted embryos. DIC images of eyes from live *tbx2b*^*+/+*^ (top panel) and *tbx2b*^*fby*^ (bottom panel) embryos (B). Quantification of SOS closure in wild type and *tbx2b*^*fby*^ mutant zebrafish eyes (C). Data are means ± SEM; one-way ANOVA with Tukey’s test: *P<0.05. Scale bars are 50 μm.

Transcriptome analyses of Gdf6a-depleted retinas have highlighted critical regulators of dorsal-ventral patterning within the zebrafish eye [31]. Using this dataset, we interrogated the superior coloboma patient exome data, and identified a variant in *TBX2* (p.Pro329His). Zebrafish *tbx2b* is expressed in the dorsal eye in a Gdf6a- and BMP-dependent manner (Fig 6A)[13]. To analyze the function of zebrafish *tbx2b* in regulating sulcus morphogenesis, we compared dorsal eye morphology between wild type embryos and *tbx2b*^*fby*^ (*from beyond*) mutants [33]. We note a statistically significant increase in the proportion of embryos displaying an open SOS in *tbx2b*^*fby*^ mutants compared to wildtype embryos at 28 hpf (Fig 6B and 6C). Such experimental results support a model in which dorsal-ventral patterning within the embryonic eye provides essential cues for morphogenesis of the SOS.

### Superior ocular sulcus functions as a conduit for superficial vasculature

**Fig 7:**
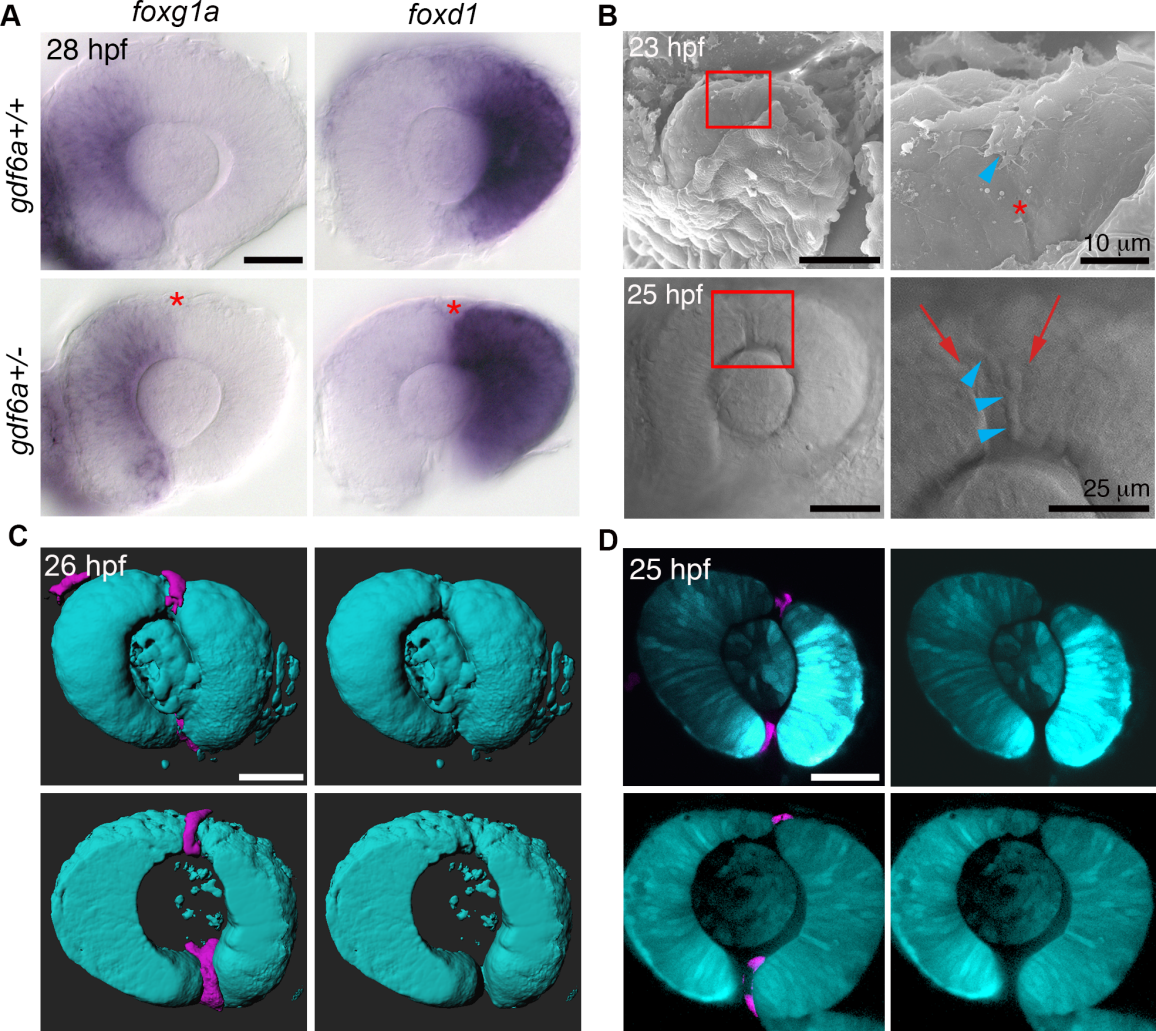
Developmental functions of the superior ocular sulcus. (A) The prominent and persistent SOS (red asterisk) present in *gdf6a*^*+/-*^ embryos aligns with the boundary between the nasal marker *foxg1a* and temporal marker *foxd1*. Note that nasal-temporal patterning is unchanged in the *gdf6a* heterozygotes (bottom row) compared to the wildtype embryos (top row). (B) SEM photographs showing the dorsal radial vessel (DRV) extending into the SOS (top row). DIC images of DRV (blue arrowheads) within a wide SOS (red arrows) (bottom row). Right panels are magnified views of boxed regions. (C-D) Surface projections (C) and single optical slices (D) from confocal images of *Tg(rx3*:*GFP; kdrl*:*mCherry)* embryos show the DRV (magenta) extending through the SOS (optic cup and lens are cyan). Scale bars are 50 μm unless otherwise noted.

The inferior fissure demarcates the boundary between nasal and temporal retinal lobes and allows for ingrowth of blood vessels into the developing eye, both of which are also logical functions for the SOS. Alignment of the SOS with naso-temporal markers was examined in *gdf6a*^*+/-*^ embryos because of their easy-to-visualize sulcus and undisturbed nasal-temporal patterning. In situ hybridization with probes for *foxg1a* (nasal retina) and *foxd1* (temporal retina) demonstrates that the expression boundaries align with the position of the sulcus. Although the SOS lies at the division between nasal and temporal retina (Fig 7A), its significance in separating retinal domains or, conversely, the role of nasal-temporal patterning in establishing the location of the sulcus remain to be tested.

Vascular inputs to the developing zebrafish eye include both the hyaloid artery that extends through the inferior fissure to form a plexus behind the lens, and the superficial vasculature that grows over the eye and encircles the lens [8,9]. The two systems are connected ventrally by the hyaloid vein. We hypothesized that the SOS forms a channel for the dorsal radial vessel (DRV, the first vessel of the superficial vasculature) as it grows over the dorsal retina and toward the lens. Indeed, SEM imaging shows a vessel extending into the SOS, and both DIC and confocal time-lapse imaging demonstrate that the nascent DRV grows through the sulcus (S3 and S9 Videos and Fig 7B–7D).

**Fig 8:**
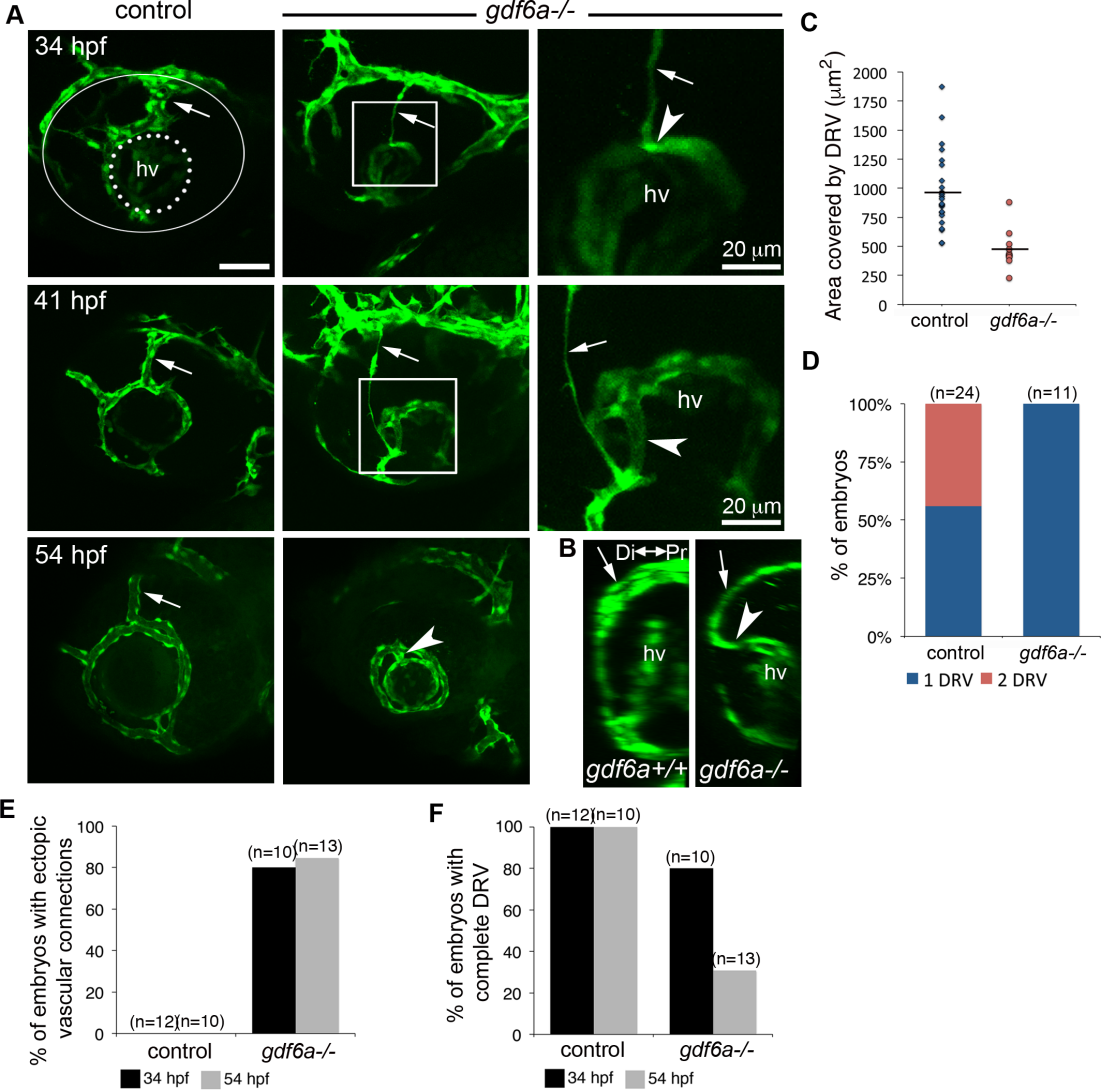
Abnormal ocular vasculature in *gdf6a* homozygous mutants. (A-B) Growing blood vessels (green) in the developing eyes of *gdf6a*^-/-^ or control (sibling) embryos are highlighted by the *kdrl*:*eGFP* transgene and shown as maximum projections of confocal z-stacks (A) or 90° lateral rotations thereof (B). Dorsal radial vessels (DRVs) are indicated by arrows. In the top left panel, the lens is outlined with a dotted line and the entire eye with a white line. The DRV forms in most *gdf6a*^*-/-*^ mutants (shown at 34 hpf), can be observed degrading in 41 hpf embryos, and is often absent by 54 hpf. Ectopic connections (arrowheads) between DRV and hyaloid vasculature (hv) are visible in *gdf6a*^*-/-*^ embryos. Right panels are enlarged views of boxed regions. (B) Laterally rotated images showing ectopic connection to hyaloid vasculature in a *gdf6a*^*-/-*^ embryo, but not in a wildtype embryo at 41 hpf. (C-D) Quantification of area and number of DRV vessel(s) in 26 hpf control and *gdf6a*^*-/-*^ embryos. (E-F) Quantification showing percentage of control and *gdf6a*^*-/-*^ embryos with an ectopic connection between the hyaloid and superficial vascular systems (E) and a complete DRV (F) as assessed at 34 and 54 hpf. n = number of embryos. Scale bar is 50 μm.

**Fig 9:**
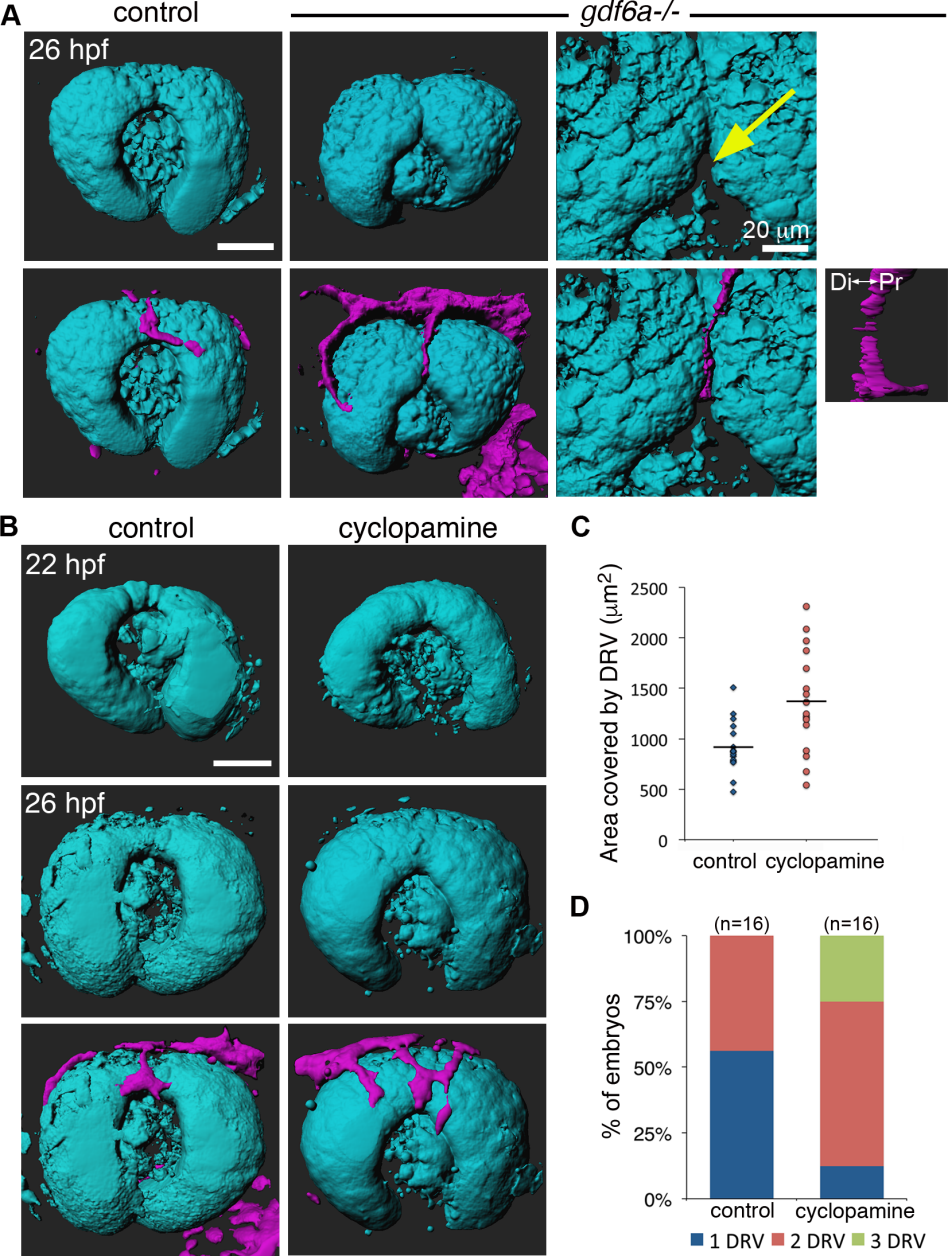
Aberrant SOS closure leads to abnormal vasculature. (A) Surface projections of 26 hpf *Tg(rx3*:*GFP; kdrl*:*mCherry)* wildtype and *gdf6a*^*-/-*^ embryos, shown without vessels (top row) and with vessels (bottom row). Last column shows expanded views of same *gdf6a*^*-/-*^ eye, highlighting the divot in the dorsal retina at the inferior edge of the superior ocular sulcus (yellow arrow). Small panel is 90° lateral rotation of vessel in adjacent panel, showing the DRV turn and extend toward the hyaloid vasculature. (B) Surface projections of *Tg(rx3*:*GFP; kdrl*:*mCherry)* embryos before (22 hpf) and after (26 hpf) DRV formation, with and without cyclopamine treatment. (C-D) Quantification of the area and number of DRV vessel(s) in control and cyclopamine-treated 26 hpf embryos. n = number of embryos. Scale bars are 50 μm unless otherwise noted. Di-Pr, distal-proximal.

If the SOS functions to direct the DRV toward the lens, then altered sulcus morphology and dynamics would be expected to modify vascular development. Since our data demonstrate that Bmp signaling regulates SOS closure, we therefore evaluated development of the superficial vasculature in embryos lacking Gdf6a. The DRV does form in *gdf6a*^-/-^ mutants and extends through the abnormally deep SOS; however, compared to control embryos, the DRV is of reduced caliber and unbranched at 26 hpf [*gdf6a*^-/-^: 0±0 branch points (n = 11) vs. siblings: 1.3±1.0 branch points (n = 24)] (Fig 8A, 8C and 8D). The *gdf6a* mutants always form a single DRV, compared to approximately half of control embryos where two DRV converge in the SOS (see control 41 hpf embryo in Fig 8A, Fig 8D). Moreover, instead of its normal course around the lens, the DRV in *gdf6a*^-/-^ embryos projects deeply and ectopically travels dorsal to the lens to connect with the hyaloid vasculature (Fig 8A, 8B and 8E). The DRV subsequently degenerates in most *gdf6a* mutants, but the ectopic vessel remains as a dorsal connection between the superficial annular vessel and the hyaloid plexus (Fig 8A, 8E and 8F). Imaging of *Tg(rx3*:*GFP;kdrl*:*mCherry)* embryos revealed that the deep sulcus in *gdf6a*^*-/-*^ mutants creates a notable divot in the optic cup immediately dorsal to the lens (Fig 9A). In all cases (n = 8), the forming ectopic vessels grew directly into this space between the dorsal edge of the lens and the retina. Given the defects observed for the DRV in Bmp-deficient embryos, we conclude that dorsal retinal patterning is necessary for superficial vascular pathfinding.

Patterning of the ventral retina is regulated by Shh [16,32], and our earlier data suggest a balance between Bmp and Shh signaling impacts SOS morphogenesis. In contrast to Bmp loss, cyclopamine inhibition of Shh signaling in wild type embryos resulted in a shallow SOS that closes early (Fig 9B), and an increased proportion of embryos with multiple DRVs spread across the dorsal retina (Fig 9B and S7 Fig). A similar change in growth of the DRV was noted previously in embryos where the Shh receptor Smoothened is non-functional [34]. In summary, disrupted dorsal-ventral patterning of the retina leads to profound alteration of the superficial vasculature.

Aberrant vasculature in *gdf6a*^*-/-*^ mutants or cyclopamine-treated embryos could result either from a direct role of the morphogens in regulating vascular pathfinding or from altered SOS dynamics. To determine whether the SOS itself directly influences growth of the superficial vasculature, we prevented the Gdf6a-dependent sulcus defects by manipulating Hedgehog signaling. Indeed, cyclopamine treatment of *gdf6a*^*-/-*^ mutants both rescues SOS closure defects and precludes ectopic connection with the hyaloid vasculature (Fig 5 and S7 Fig). Similarly, loss of Gdf6 rescues the DRV overgrowth phenotype observed in cyclopamine-treated embryos (S7 Fig). Therefore, the data support a model in which proper SOS formation and closure are necessary for DRV pathfinding.

## Discussion

In this manuscript, we classify superior coloboma as a separate disease with a developmental origin distinct from, but comparable to, inferior coloboma. Eight patients display gaps in tissues of the superior eye, including retina, lens, and iris. We demonstrated the existence of a transient dorsal groove in vertebrate eye development that is conserved amongst fish, chick, newt and mouse. Failure to close the superior ocular sulcus can result in adult zebrafish displaying a phenotype that resembles superior coloboma. Furthermore, it supports the evolutionary conservation of the SOS amongst vertebrates, an evolutionary distance of some 450 million years.

There are rare reports in the scientific literature of patients with “atypical” coloboma [35–39], ocular anomalies contrasting with the position of the known inferior embryonic fissure. The vast majority of such cases (macular coloboma, aniridia, or nasally/temporally oriented iris coloboma) are unlikely to arise from defects of sulcus closure. However, at least two of the described atypical coloboma patients display iris colobomata with a superior orientation [38,39]. Although the embryonic mechanism was originally considered anomalous, our identification of the SOS provides a likely explanation for the unusual coloboma identified in these two patients.

Exome sequencing of our superior coloboma patients identified rare variants in the genes encoding the type 1 BMP receptor and transcription factor T-box 2. In the absence of multigenerational pedigrees of affected patients, we are unable to causally link such variants to the incidence of disease. However, the connection between Bmp signaling and inferior fissure morphogenesis is well established. Indeed, variants in *GDF6* (*BMP13*), *BMP4*, and *SMOC1* are linked to inferior coloboma and microphthalmia [5,6,40–42]. Furthermore, zebrafish, *Xenopus*, chick, and mouse studies have demonstrated a key role for Bmp signaling in optic cup morphogenesis, apoptosis, proliferation, and dorsal-ventral eye patterning [11–13,40,41,43–45]. Consistently, abrogating Bmp signaling either by DMH1 treatment or loss of Gdf6a results in profound SOS closure defects. Beyond the *gdf6a* homozygous mutant phenotype, we also detected a partially penetrant sulcus closure defect in the otherwise morphologically normal *gdf6a* heterozygotes, arguing that the sulcus is particularly sensitive to the levels of Bmp signaling. Further, loss of Tbx2b function in zebrafish *fby* mutants leads to comparable aberrations in SOS morphogenesis. Such data, taken together with the detrimental nature of the patient *BMPR1A* variant, support a model whereby Bmp signaling modulates SOS closure via regulation of target genes such as *tbx2*.

Research on ocular Bmp signaling defines roles in regulating eye precursor cell number, apoptosis, proliferation, and dorsal-ventral gene expression [12,13,17,31,41,44,46,47]. However, apoptotic cell populations are not localized to the SOS, and proliferative defects are present only after SOS closure [31,47]. Furthermore, we note that *gdf6a*^*+/-*^ heterozygotes display aberrant sulcus closure, yet lack apoptotic or proliferative defects. In contrast, *gdf6a*^*+/-*^ heterozygotes display detectable alterations to dorsal-ventral gene expression, providing a correlation between patterning and SOS closure defects. To further test the role of dorsal-ventral patterning in sulcus dynamics, we asked whether rescue of the patterning defects in *gdf6a*^*-/-*^ mutants would also promote SOS closure. Given the expansion of inferior markers into the superior retina of *gdf6a*^*-/-*^ mutants [12,13], and the rescue of SOS defects with Shh inhibition, we conclude that the aberrant closure of the SOS in Gdf6- and Tbx2-depleted embryos is linked to dorsal-ventral patterning defects of the vertebrate eye.

The identification of a patient with two variants in *CYP1B1* prompted us to carefully examine retinoid signaling in SOS closure. A role in ocular morphogenesis is well established for the retinoid signaling pathway, with mutations in the RA synthesis gene *ALDH1A3* known to cause inferior coloboma [48]. Furthermore, RA regulates proliferation and migration of periocular mesenchyme (POM), a neural crest- and mesoderm-derived cell population that modulates inferior fissure closure. The inability of extensive zebrafish experiments to reveal a role for *cyp1b1* in SOS closure, even in the context of Gdf6a deficiency (S8 Fig) may reflect the greater complexity of the family of retinoid synthesis enzymes in humans and their distinct expression patterns compared to zebrafish. Given the proximity of RA signaling to the SOS and known roles for RA in regulating morphogenesis in other systems, it remains plausible that RA signaling contributes to the causality of human superior coloboma.

Eye morphogenesis and patterning are dependent on multiple signaling pathways, in addition to Bmp and RA. For example, overexpression of the Wnt inhibitor Dkk1 results in loss of dorsal ocular gene expression [49], and mutation of the Wnt receptor *FZD5* (thought to function as a receptor for both canonical and non-canonical Wnts) causes inferior coloboma [50]. In examining the prioritized list of rare variants identified in superior coloboma patients, we note rare variants in *NKD1*, *CELSR2*, *FZD4*, *SCRIB*, and *WNT9B* (components of canonical or non-canonical Wnt pathways). The rare *TSC2* (Tuberous Sclerosis Complex 2/Tuberin) variant in patient #1 plausibly implicates other cellular mechanisms in the induction of superior coloboma. TSC2 complexes with TSC1 to regulate the mTOR signaling pathway [51], and loss of either gene leads to unregulated cell growth and proliferation.

The rare incidence of superior coloboma argues that the disorder is unlikely caused by simple, single-gene inheritance. Rather, a model incorporating multi-gene inheritance or incomplete penetrance is more plausible. Seven of the eight patients with superior coloboma in the current study display unilateral disease, also a common characteristic of inferior coloboma [52]. The highly penetrant defects found in zebrafish *gdf6a* mutant larvae, which only infrequently result in an adult superior coloboma phenotype (Fig 4F), are consistent with an impressive ability of the developing eye to recover from embryonic defects. However, the absence of an obvious coloboma does not preclude abnormal SOS morphogenesis generating subtler abnormalities, such as vascular misrouting. Although defining the relative contribution of heritability and environment is challenging, other disorders offer potential insight. Characterized by appreciable globe enlargement, high myopia represents an ocular disorder with substantial genetic and environmental components, where unilateral cases account for up to one third of the total [53]. Anisometropia represents a second example of an asymmetric developmental ocular phenotype [54], and the pattern apparent in the current cases (Fig 1) corresponds with such examples.

**Fig 10:**
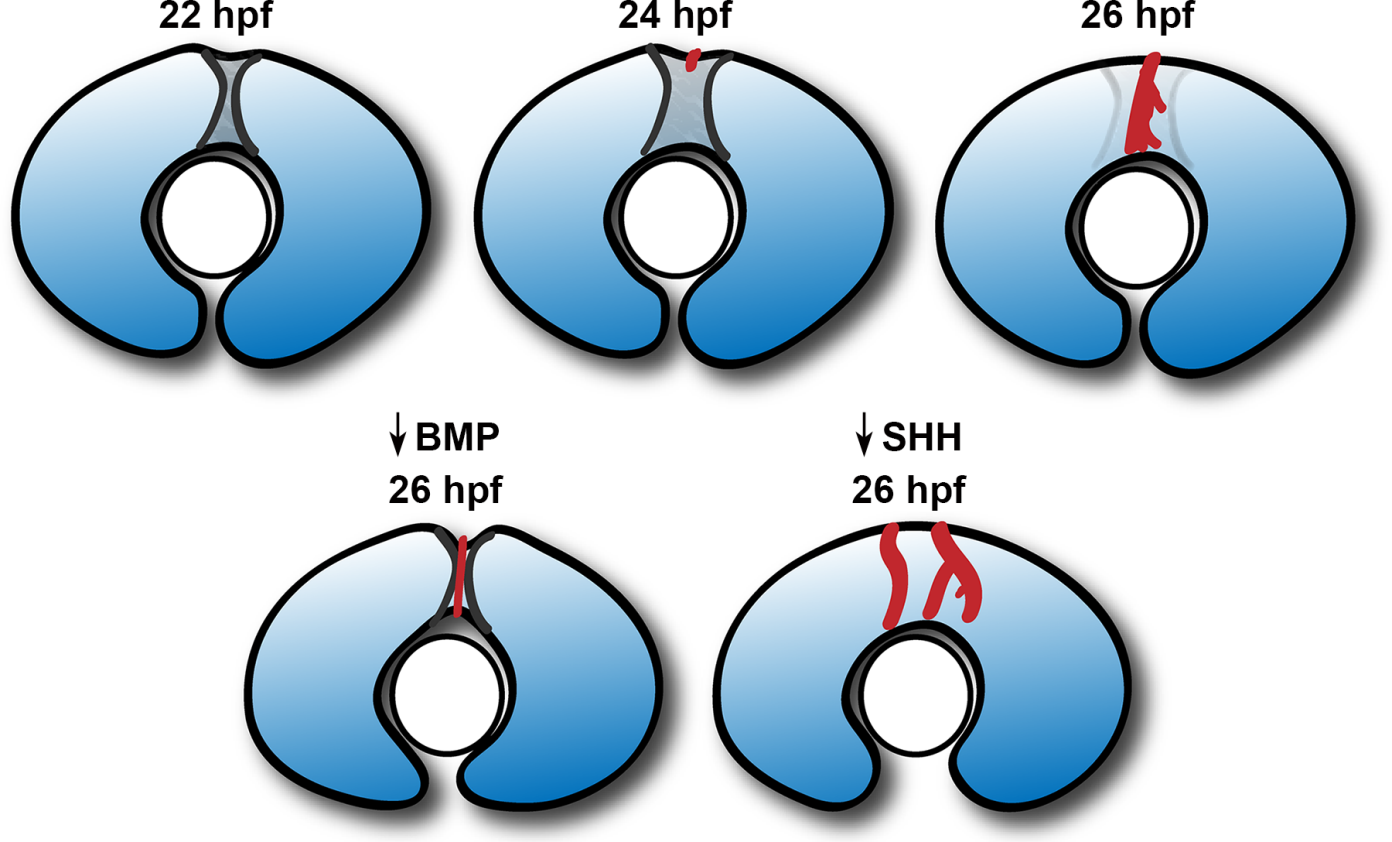
Model of superior ocular sulcus morphogenesis and function. The superior ocular sulcus appears as a narrow groove in the dorsal retina soon after optic cup formation (22 hpf), and subsequently becomes wider (24 hpf). The DRV grows through the wide sulcus as it travels across the dorsal retina towards the lens (24–26 hpf). If BMP signaling is reduced, the sulcus persists as a deep and narrow structure, through which the DRV still travels. However, in low BMP conditions, the DRV has a thin and unbranched morphology as it traverses the deeper fissure, and then enters the divot at the inferior edge of the sulcus and forms an ectopic connection with the hyaloid vessels. If Sonic Hedgehog signaling is reduced, the SOS is absent at the time of DRV growth, resulting in the formation of more DRV vessels spread across the dorsal retina.

The parallels with the inferior ocular fissure, which provides a passageway for the hyaloid vasculature [7,9,55], are strong. The close coordination between the development of both structures is highlighted by the ability of the dilated hyaloid vein in zebrafish *lmo2* mutants to disrupt fissure closure and cause inferior coloboma [56]. The tight association between the superficial vasculature’s DRV and the SOS provides convincing evidence that the SOS serves a similar retinal vascular guidance function. While developing blood vessels follow guidance cues in the same manner as growing axons [57], our data argues that the physical landscape of a tissue can also direct angiogenesis. First, the DRV in wildtype embryos travels directly through the SOS to reach the lens, whereas only a thin and unbranched DRV grows through the particularly deep sulci of *gdf6a*^*-/-*^ mutants. Second, the shallow or absent SOS in cyclopamine-treated embryos correlates with the appearance of multiple DRVs spread across the dorsal retina. Finally, the divot above the lens in *gdf6a*^*-/-*^ mutants aligns with the position of the ectopic connection between hyaloid and superficial vasculature. Taken together, these data support a model in which the SOS provides a path for directing and restraining DRV growth (Fig 10).

Here, we have characterized a previously unrecognized developmental structure with a significant disease connection. Further studies will be needed to discern the exact mechanisms of sulcus formation and resolution, and to more deeply analyze the causes of superior coloboma.

## Materials and methods

### Ethics statement

All experiments were conducted in accordance with the guidelines provided by the institutional IACUC. The research on chick and mouse was performed at the University of Texas and approved by their institutional AC committee (#2015–00089). The research on zebrafish was performed at the University of Alberta and approved by ACUC, Biosciences (#0082) and ACUC, Faculty of Medicine (#1476). Anesthesia and euthanasia were performed with MS-222.

### Exome sequencing

Whole exome sequencing was performed on genomic DNA from each proband (#1 - #5) as part of FORGE Canada Consortium at the McGill University and Génome Québec Innovation Centre. Exome target enrichment was performed using the Agilent SureSelect 50Mb (V3) All Exon Kit and sequencing was performed on the Illumina HiSeq 2000, multiplexing three samples per lane. The mean coverage of coding sequence regions, after accounting for duplicate reads was greater than 70x. WES data was analyzed by performing alignment with BWA, duplicate read removal with Picard, local indel realignment with GATK, variant calling with SAMtools, and annotation with Annovar and custom scripts [58]. Subsequently exome sequencing was repeated commercially (Beijing Genomics Institute). In parallel, array CGH was performed to identify any causative copy number variations (CNV) using an Affymetrix cytoscan HD array that comprises approximately 1,800,000 CNV and 700,000 genotyping probes. Within patients #1–5, we identified 783, 843, 942, 708, and 721 rare (<1%) non-synonymous and stop-gain/loss variants, respectively. By filtering such variants using Mutation Taster (score >0.95), patients #1–5 contain 163, 155, 139, 112, and 148 higher probability variants, respectively. Subsequent prioritization included literature searches associating genes with ocular function and zfin.org examination of in situ expression patterns within the developing eye at 18–24 hpf, yielding a restricted subset of high priority variants in each proband (S1 Table).

### Zebrafish husbandry and in situ hybridization

Zebrafish were cared for according to standard protocols, and embryos grown in embryo media at 28.5°C and staged appropriately. Zebrafish embryos grown past 24 hours post fertilization (hpf) were treated with 0.004% 1-phenyl 2-thiourea (PTU; Sigma-Aldrich, St. Louis, MO) to prevent pigment formation. The AB strain of wild-type (WT) fish, *Tg(rx3*:*GFP)*, *Tg(kdrl*:*eGFP)*^*la116*^, *and Tg(kdrl*:*mCherry)*^*ci5*^ transgenic lines [59–61], and *cyp1b1* (see below), *gdf6a*^s327^ [13], and *tbx2b*^*fby*^ [33] mutant lines were used. The *gdf6a*^s327^ mutation encodes a S55X truncation producing a 54 amino acid peptide lacking the mature domain. The *tbx2b*^*fby*^ mutation is a point mutation resulting in a T>A substitution, resulting in a premature stop codon within the T-box sequence. In situ hybridization (ISH) was performed as described previously [12] with embryos fixed overnight at 4°C in 4% paraformaldehyde (PFA) and permeabilized by incubation in 10 μg/mL Proteinase K for 20 minutes for 28 hpf embryos and 1 min for 5.3 hpf (50% epiboloy) embryos. Following in situ hybridization, eyes from 28 hpf embryos were removed and mounted under a coverslip in 70% glycerol, and then photographed using a Zeiss Axioimager Z1 compound microscope and an Axiocam HR digital camera (Carl Zeiss Microscopy, LLC). 5.3 hpf embryos were imaged using an Olympus stereoscope and a Qimaging micropublisher camera.

### Chick and mouse embryos

For the chick studies, fertilized Leghorn eggs (Texas A&M, Bryan, TX) were incubated at 38°C in a humidified forced-draft incubator. Chick embryos were staged according to Hamburger and Hamilton [62] and Swiss Webster mice were collected at E10.5

### Immunocytochemistry and imaging of chick and mouse

Immunohistochemistry was performed as previously described [63]. Chick embryos were stained with antibodies against Laminin-1 (#3HL1; Developmental Hybridoma Studies Bank, Iowa; Conc: 1:250), whereas for mouse Laminin alpha 1 stains, we utilized a different antibody (Sigma L9393). Alexa-Fluor conjugated Goat anti-Rabbit IgG (#411008; Life Technologies,: Conc: 1:250) was used for fluorescent detection [64]. Antibodies used in the current study were validated for use in chicks in previous studies [64,65]. DAPI staining was used for detecting nuclei. Confocal images were obtained with an Olympus IX51 spinning disc microscope and data analyses carried out with Slidebook Pro (3I, CO). Images are presented as single 0.5–0.8 μm thick optical sections. The position in the dorsal-ventral plane is based on the acquisition of multiple serial sections and respective alignment to those sections (just ventral) that contain lens tissue.

### Morpholino injections

A *gdf6a* Morpholino (5’-GCAATACAAACCTTTTCCCTTGTCC-3’) was used to block splicing at the exon 1–intron 1 boundary of the *gdf6a* pre-mRNA transcript [41]. 5–10 ng were injected into one-cell stage wildtype zebrafish embryos.

### TALEN mutagenesis

TALEN mutagenesis constructs targeting the Cyp1b1 cytochrome P450 domain (nts 924–977) were created by Golden Gate cloning [66]. The target region was TTCGGGGCCAGTCAAGACACtctgtctacagctCTCCAGTGGATCATCCTGCTA, with the spacer region shown in small letters. 100 pg of RNA for each TAL construct were injected into one-cell stage zebrafish embryos and the offspring were screened for mutations by HRM. The 13 bp deletion causes a frameshift at aa317 (p.Cys317SerfsX23), followed by a stop codon at aa340. The wildtype protein is 526aa.

### Zebrafish genotyping

The offspring of *gdf6a* heterozygous incrosses were genotyped by high resolution melt (HRM) temperature analysis performed on genomic DNA extracted in 10 μL of 50 mM NaOH (95°C, 10 minutes) and neutralized with 2 μL Tris-HCl, pH 8.0. PCR was performed using primers optimized for HRM (GCGTTTGATGGACAAAGGTC; CCGGGTCCTTAAAATCATCC) and Qiagen Master Mix on a Qiagen Rotor Gene Q qPCR machine (Qiagen). Conditions for amplification were 1 cycle at 95°C for 5 min, 40 cycles of 95°C for 10 seconds, 55°C for 30 seconds, followed by HRM ramp from 70–90°C, 0.1°C per step. Fish carrying the *cyp1b1* mutation were HRM genotyped using primers CCATCTCAGATATTTTCGGGG and GTTATTTACCTGACAAGTAGCAG and a 52°C annealing temperature for amplification. Results were analyzed via Qiagen software v2.02 (Qiagen) and variants initially confirmed by Sanger sequencing. *tbx2b*^*fby*^ mutants were genotyped by PCR followed by MseI restriction digest. Genomic DNA was extracted as above and diluted 10X for use as template. PCR was performed with Ex Taq DNA Polymerase (TaKaRa Bio Inc.) using the following primers: Forward-TGTGACGAGCACTAATGTCTTCCTC; Reverse-GCAAAAAGCATCGCAGAACG. Conditions for amplification were 1 cycle at 94°C for 2 min, 40 cycles of 94°C for 15 seconds, 58°C for 15 seconds, and 72°C for 20 seconds, followed by 1 cycle at 72°C for 3 min. The PCR products were then digested with MseI (NEB) for two hours and analyzed via gel electrophoresis using a 3% agarose gel.

### Immunocytochemistry of Zebrafish embryos

For Laminin staining, whole embryos fixed in 4% PFA were permeabilized with ice-cold acetone (7 min), washed in water (5 min), and PBS with 0.5% Tween-20 (PBST, 5 min). Embryos were then treated for 5 min with 10 μg/mL Proteinase K in PBST, washed four times in PBST, blocked for 1 hour at room temperature in 5% normal goat serum and 2% BSA, and then incubated overnight at 4°C in block plus rabbit anti-Laminin primary antibody (1/100, L-9393, Sigma-Aldrich). After washing, embryos were incubated at room temperature for two hours in goat anti-rabbit Alexa Fluor 488 or 568 secondary antibody (1/1000, Molecular Probes). All embryos were washed 4 times for 10 minutes in PBST after primary and secondary antibody incubations. Eyes were removed and mounted on slides in 70% glycerol, or were imaged as whole embryos mounted in 1% low melting temperature agarose.

### Scanning electron microscopy

Embryos at 22 hpf from AB (wildtype) and *gdf6a*^+/-^ incrosses were fixed overnight in 2.5% Glutaraldehyde; 2% Paraformaldehyde. After washing in 0.1M phosphate buffer, embryos were gradually dehydrated in ethanol, transferred to Hexamethyldisilazane (HMDS; Electron Microscopy Sciences) and left to dry overnight. Embryos were then mounted on SEM stubs, sputter coated with Au/Pd using a Hummer 6.2 Sputter Coater (Anatech), and imaged on a XL30 scanning electron microscope (FEI) operating at 20 kV.

### Construction and injection of BMPR1A constructs

The full coding domain of human *BMPR1A* mRNA (Ensembl transcript ID: ENST00000372037) was cloned into pCS2+. Using site-directed mutagenesis, the Q233D mutation was created to make caBMPR1a [28] and the patient mutation, G1412A, was subsequently added to create R471H-caBMPR1a, with sequences confirmed by Sanger sequencing. The constructs, which were identical except for the patient mutation, were linearized (NotI, NEB) and mRNA generated using the SP6 mMessage mMachine kit (Ambion). mRNA was purified using YM-50 Microcon columns (Amicon, Millipore) and concentration determined through spectrophotometry. The mRNA, which was newly synthesized for each round of injections, was diluted with DEPC-treated water, and embryos were injected with 25 pg of RNA at the single-cell stage. Both injections and analysis were performed in a blinded fashion.

### Quantitative real-time PCR (qRT-PCR)

Primers were validated prior to the experiment, as previously described [67]. Endogenous control primers (*elongation factor 1a; ef1a*), previously used [67], were chosen from the Universal Probe Library Assay Design Centre for Zebrafish (Roche). Primer sequences for *BMPR1A* are as follows: F-CGTGTTCAAGGACAGAATCTGG; R-AAAGGCAAGGTATCCTCTGGTG. RNA was isolated from 40 embryos per group at the 256-cell stage using RNAqueous (Ambion), first-strand cDNA synthesis was performed using AffinityScript qPCR cDNA Synthesis (Agilent) and qRT-PCR was performed as described previously [67].

### Pharmacological treatments

For drug treatments, embryos were transferred at 10 hpf or 18 hpf into 35 mm dishes containing 5 mL embryo media plus 0.05–0.2 μM DMH1 (Sigma-Aldrich), 1–5 μM N,N-diethylaminobenzaldehyde (DEAB; Sigma-Aldrich), 0–10 μM cyclopamine (Sigma-Aldrich), or an equivalent volume of vehicle (DMSO or ethanol). For each experiment, 15 embryos were added to each dish and two dishes were used for each treatment (total of 30 embryos). For analysis of SOS presence, embryos were grown at 28.5°C to 28 hpf, treatment conditions were blinded and the embryos were analyzed under a Zeiss Discovery V8 80x stereoscope for the presence of the SOS. Alternatively, live or fixed embryos at 22–54 hpf were mounted in 1% low melting temperature agarose for imaging.

### Statistics

Two-factor analysis was done by Students *t* test. Multivariable analysis was performed by two-tailed, one or two-factor ANOVA with Tukey posthoc test. *P<0.05, **P<0.01, ***P<0.001.

### Imaging of zebrafish embryos

Fixed or anaesthetized live transgenic embryos were mounted laterally in a 35mm dish in 1% low-melting temperature agarose and imaged using a Zeiss W Plan-Apochromat 20x/1.0 water immersion objective and a Zeiss LSM700 laser scanning unit mounted on a Zeiss Axioimager Z1 compound microscope. Z-stacks were made by taking optical slices at intervals of 2–3 μm for a total of ~60 μM, and maximum projections or surface projections were created from the resulting stacks using either ZEN (Carl Zeiss) or Imaris (Bitplane) software.

All DIC images were taken on an Axiocam HR digital camera mounted on a Zeiss Axioimager Z1 compound microscope. Photos and videos were annotated, assembled and processed for brightness and contrast in Adobe Photoshop software.

### Members of FORGE Canada Consortium

FORGE Canada Consortium: Finding of Rare Disease Genes in Canada; Steering Committee: Kym Boycott (leader; University of Ottawa), Jan Friedman (co-lead; University of British Columbia), Jacques Michaud (co-lead; Université de Montréal), Francois Bernier (University of Calgary), Michael Brudno (University of Toronto), Bridget Fernandez (Memorial University), Bartha Knoppers (McGill University), Mark Samuels (Université de Montréal), Steve Scherer (University of Toronto).

## Supporting information

S1 Table Superior coloboma patient information. (DOCX)

S2 Table Genetic variants in superior coloboma patients. Exome sequencing of superior coloboma patients identified rare variants (<1% frequency in general population) that were subsequently prioritized on the basis of high Mutation Taster score (>0.95) and prior association with ocular gene expression or function.
(XLSX)

S1 Fig Genetic variants identified in bilateral superior coloboma patient. (A) Diagram of the human CYP1B1 protein, with the compound heterozygous mutations carried by patient#2 indicated. (B) Diagram of the human BMPR1A protein showing rare variant present in patient#2. (C) Alignment illustrating the evolutionary conservation of the BMPR1A protein kinase domain. The altered residue (p.**R**471H) is depicted in bold, with invariant residues denoted by *.
(TIF)

S2 Fig Superior ocular sulcus in chick. (A) 3D Model of the eye depicting where the eye was sectioned to create the serial horizontal sections shown in B. B) Serial cryostat sections of a chick HH16 stage eye stained with DAPI (blue) and α-Laminin antibody (green). First three sections are dorsal to the lens and third one is through the lens. (C) Tangential section of HH16 chick eye labeled with DAPI (blue) and α-Laminin antibody (green). D, dorsal; V, ventral; Di, distal; Pr, proximal; HH, Hamburger Hamilton. Red asterisks indicate superior ocular sulcus. Scale bar is 50 μm.
(TIF)

S3 Fig Superior ocular sulcus in newt. Scanning electron microscopy images of newt (*Taricha tarosa*) ocular development. Panels on left display SEM images of stage 34 embryos after partial dissection of surface tissues. Panels on right show slightly older embryos (stage 36–37), with vasculature intact in the stage 36 example. Red asterisks indicate superior ocular sulcus.
(TIF)

S4 Fig Dynamics of the zebrafish superior ocular sulcus. (A) Time-lapse images showing lateral views of the eye of a *Tg(rx3*:*GFP)* embryo. The superior ocular sulcus appears as a narrow groove across the dorsal retina at ~20 hpf (red asterisk), becomes wider by 24 hpf (red arrows) and disappears after 26 hpf. (B) Timing of SOS as viewed under a stereomicroscope. The wide and shallow phase is not visible by stereomicroscope, so the red bars indicate the percentage of embryos with a narrow and distinct sulcus.
(TIF)

S5 Fig Reduced RA signaling does not impair closure of the superior ocular sulcus. (A) Lateral view of a 28 hpf zebrafish eye following in situ hybridization for *cyp1b1*. Note that expression extends into the dorsal eye. (B) Quantification of open SOS in 28 hpf embryos from *cyp1b1*^*+/-*^ incrosses treated from 10 hpf with control solution or the Aldh inhibitor DEAB. N = 3 experiments, n = number of embryos. Data are means ± SEM. Statistics is two-way ANOVA with Tukey's test. Scale bar is 50 μm. ns, not significant.
(TIF)

S6 Fig Human variant in BMPR1A reduces protein function. (A) One cell-stage zebrafish embryos were injected with *caBMPR1A* or *R471H-caBMPR1A* mRNA, and assessed at 24 hpf for morphological abnormalities by categorization according to the pictures shown. (B) Graph showing percentage of embryos injected with *caBMPR1A* (n = 22 embryos) or *R471H-caBMPR1A* (n = 23 embryos) that fit into each category of morphological abnormality. (C) qPCR showing equal amounts of injected RNA for each condition. Statistics is two-tailed *t* test.
(TIF)

S7 Fig Interaction between Hedgehog and Bone Morphogenetic Protein signaling in formation of the dorsal radial vessel. Maximum projection confocal images of 34 hpf eyes from *Tg(kdrl*:*eGFP);gdf6a*^*+/+*^ and *Tg(kdrl*:*eGFP);gdf6a*^*-/-*^ zebrafish embryos following treatment with control solution or 10 μM cyclopamine from 10 hpf. Blood vessels fluoresce green and the eye is outlined by dotted lines. DRV is indicated by arrow. Ectopic connection between superficial and hyaloid vasculatures indicated by arrowhead. Top row, right two panels are two examples of vessel overgrowth phenotype in cyclopamine-treated wildtype embryos. Bottom row, middle and right panels show the eyes of cyclopamine-treated *gdf6a*^*-/-*^ embryos that either failed to form a DRV (n = 3/6 embryos) or grew a simple DRV that did not make an ectopic connection to the hyaloid vasculature (n = 3/6 embryos), respectively. hv, hyaloid vasculature. Scale bar is 50 μm.
(TIF)

S8 Fig Retinoic acid signaling and the superior ocular sulcus. (A) Lateral views of eyes from 28 hpf zebrafish embryos that are *gdf6a*^*+/+*^, *gdf6a* ^*+/-*^, or *gdf6a*^*-/-*^ and have been processed for *in situ* hybridization. The top two rows show expression of the retinoic acid-synthesis genes *cyp1b1* and *aldha1a2*. The bottom row shows expression of *GFP* in transgenic zebrafish carrying a reporter for RA signaling [*Tg(12xRARE*:*GFP)*] and are also *gdf6a*^*+/+*^, *gdf6a* ^*+/-*^, or *gdf6a*^*-/-*^. Note reduced RA signaling in the superior retina of *gdf6a* ^*+/-*^ and *gdf6a*^*-/-*^ embryos. (B) Graph showing no effect of retinoic acid treatment on SOS closure. Embryos from *gdf6a*^*+/-*^ incrosses were grown from 10 hpf in control media, 5 nM retinoic acid, or 10 nM retinoic acid, and assessed at 28 hpf for an open SOS. (C) Graph showing no effect of the *cyp1b1* mutation on SOS closure in *gdf6a* heterozygotes. *gdf6a*^*+/-*^*;cyp1b*^*+/-*^ fish were crossed to wildtype or *cyp1b*^*-/-*^ fish and the percentage of embryos with an open SOS was assessed at 28 hpf. n = number of embryos, N = 2 (B) or 3 experiments (C). Data are means ± SEM. ns, not significant
(TIF)

S1 Video Superior ocular sulcus appears after optic cup and lens formation. Red arrow points to SOS. Time stamp is hrs:mins. D, dorsal; V, ventral.
(MP4)

S2 Video Formation of the optic cup. Live imaging of the developing eye of a *Tg(rx3*:*GFP)* embryo, lateral view. Video was started at 18 hpf.
(MP4)

S3 Video Superior ocular sulcus transitions from narrow to wide. Red arrows define lateral edges of SOS. Asterisk marks growing dorsal radial vessel. Time stamp is hrs:mins. D, dorsal; V, ventral.
(MP4)

S4 Video Superior ocular sulcus does not close by epithelial fusion, lateral view. Lateral view, surface projection of the eye of a *Tg(rx3*:*GFP)* embryo, starting at 22 hpf. D, dorsal; V, ventral.
(MP4)

S5 Video Superior fissure ocular sulcus does not close by epithelial fusion, dorsal view. Dorsal view, cut surface projection of the eye of a *Tg(rx3*:*GFP)* embryo, starting at 22 hpf (same eye as in S4 Video). Di, distal; Pr, proximal.
(MP4)

S6 Video Morphology of superior ocular sulcus in a wildtype embryo. Surface projection made from a confocal z-stack through the dorsal eye of a 22 hpf *Tg(rx3*:*GFP)* embryo.
(MP4)

S7 Video Morphology of superior ocular sulcus in a *gdf6a* heterozygous embryo. Surface projection made from a confocal z-stack through the dorsal eye of a 22 hpf *Tg(rx3*:*GFP)* embryo.
(MP4)

S8 Video Morphology of superior ocular sulcus in a *gdf6a* homozygous mutant embryo. Surface projection made from a confocal z-stack through the eye of a 22 hpf *Tg(rx3*:*GFP)* embryo.
(MP4)

S9 Video The dorsal radial vessel grows through the superior ocular sulcus. Surface projections made from time lapse confocal z-stacks through the eye of a live *Tg(kdrl*:*mCherry;rx3*:*GFP)* embryo, starting at 24 hpf. mCherry expression in the blood vessels is shown in magenta, GFP in the eye and lens is cyan. Scale bar is 50 μm.
(MP4)

## References

[1] Bazin-Lopez N, Valdivia LE, Wilson SW, Gestri G. Watching eyes take shape. Curr Opin Genet Dev. 2015;32: 73–79. doi: 10.1016/j.gde.2015.02.004 25748250 10.1016/j.gde.2015.02.004 PMC4931046

[2] Fuhrmann S. Eye morphogenesis and patterning of the optic vesicle. Curr Top Dev Biol. 2010;93: 61–84. doi: 10.1016/B978-0-12-385044-7.00003-5 20959163 10.1016/B978-0-12-385044-7.00003-5 PMC2958684

[3] Chang L, Blain D, Bertuzzi S, Brooks BP. Uveal coloboma: clinical and basic science update. Curr Opin Ophthalmol. 2006;17: 447–470. doi: 10.1097/01.icu.0000243020.82380.f6 16932062 10.1097/01.icu.0000243020.82380.f6

[4] Onwochei BC, Simon JW, Bateman JB, Couture KC, Mir E. Ocular colobomata. Surv Ophthalmol. 2000;45: 175–194. 11094243 10.1016/s0039-6257(00)00151-x

[5] Williamson KA, FitzPatrick DR. The genetic architecture of microphthalmia, anophthalmia and coloboma. Eur J Med Genet. 2014;57: 369–380. doi: 10.1016/j.ejmg.2014.05.002 24859618 10.1016/j.ejmg.2014.05.002

[6] Gregory-Evans CY, Williams MJ, Halford S, Gregory-Evans K. Ocular coloboma: a reassessment in the age of molecular neuroscience. J Med Genet. 2004;41: 881–891. doi: 10.1136/jmg.2004.025494 15591273 10.1136/jmg.2004.025494 PMC1735648

[7] Saint-Geniez M, D’Amore PA. Development and pathology of the hyaloid, choroidal and retinal vasculature. Int J Dev Biol. 2004;48: 1045–1058. doi: 10.1387/ijdb.041895ms 15558494 10.1387/ijdb.041895ms

[8] Kaufman R, Weiss O, Sebbagh M, Ravid R, Gibbs-Bar L, Yaniv K, et al Development and origins of Zebrafish ocular vasculature. BMC Dev Biol. 2015;15: 18 doi: 10.1186/s12861-015-0066-9 25888280 10.1186/s12861-015-0066-9 PMC4406013

[9] Kitambi SS, McCulloch KJ, Peterson RT, Malicki JJ. Small molecule screen for compounds that affect vascular development in the zebrafish retina. Mech Dev. 2009;126: 464–477. doi: 10.1016/j.mod.2009.01.002 19445054 10.1016/j.mod.2009.01.002 PMC2775549

[10] Chambers D, Wilson L, Maden M, Lumsden A. RALDH-independent generation of retinoic acid during vertebrate embryogenesis by CYP1B1. Dev Camb Engl. 2007;134: 1369–1383. doi: 10.1242/dev.02815 17329364 10.1242/dev.02815

[11] Behesti H, Holt JKL, Sowden JC. The level of BMP4 signaling is critical for the regulation of distinct T-box gene expression domains and growth along the dorso-ventral axis of the optic cup. BMC Dev Biol. 2006;6: 62 doi: 10.1186/1471-213X-6-62 17173667 10.1186/1471-213X-6-62 PMC1764729

[12] French CR, Erickson T, French DV, Pilgrim DB, Waskiewicz AJ. Gdf6a is required for the initiation of dorsal-ventral retinal patterning and lens development. Dev Biol. 2009;333: 37–47. doi: 10.1016/j.ydbio.2009.06.018 19545559 10.1016/j.ydbio.2009.06.018

[13] Gosse NJ, Baier H. An essential role for Radar (Gdf6a) in inducing dorsal fate in the zebrafish retina. Proc Natl Acad Sci U S A. 2009;106: 2236–2241. doi: 10.1073/pnas.0803202106 19164594 10.1073/pnas.0803202106 PMC2650138

[14] Kruse-Bend R, Rosenthal J, Quist TS, Veien ES, Fuhrmann S, Dorsky RI, et al Extraocular ectoderm triggers dorsal retinal fate during optic vesicle evagination in zebrafish. Dev Biol. 2012;371: 57–65. doi: 10.1016/j.ydbio.2012.08.004 22921921 10.1016/j.ydbio.2012.08.004 PMC3455121

[15] Lupo G, Gestri G, O’Brien M, Denton RM, Chandraratna RAS, Ley SV, et al Retinoic acid receptor signaling regulates choroid fissure closure through independent mechanisms in the ventral optic cup and periocular mesenchyme. Proc Natl Acad Sci U S A. 2011;108: 8698–8703. doi: 10.1073/pnas.1103802108 21555593 10.1073/pnas.1103802108 PMC3102374

[16] Sasagawa S, Takabatake T, Takabatake Y, Muramatsu T, Takeshima K. Axes establishment during eye morphogenesis in Xenopus by coordinate and antagonistic actions of BMP4, Shh, and RA. Genes N Y N 2000. 2002;33: 86–96. doi: 10.1002/gene.10095 12112877 10.1002/gene.10095

[17] Valdivia LE, Lamb DB, Horner W, Wierzbicki C, Tafessu A, Williams AM, et al Antagonism between Gdf6a and retinoic acid pathways controls timing of retinal neurogenesis and growth of the eye in zebrafish. Dev Camb Engl. 2016;143: 1087–1098. doi: 10.1242/dev.130922 26893342 10.1242/dev.130922 PMC4852494

[18] Nordquist D, McLoon SC. Morphological patterns in the developing vertebrate retina. Anat Embryol (Berl). 1991;184: 433–440. 1741476 10.1007/BF01236049

[19] Schmitt EA, Dowling JE. Early retinal development in the zebrafish, Danio rerio: light and electron microscopic analyses. J Comp Neurol. 1999;404: 515–536. 9987995

[20] Faiq MA, Dada R, Qadri R, Dada T. CYP1B1-mediated Pathobiology of Primary Congenital Glaucoma. J Curr Glaucoma Pract. 2015;9: 77–80. doi: 10.5005/jp-journals-10008-1189 26997841 10.5005/jp-journals-10008-1189 PMC4779945

[21] Das BC, Thapa P, Karki R, Das S, Mahapatra S, Liu T-C, et al Retinoic acid signaling pathways in development and diseases. Bioorg Med Chem. 2014;22: 673–683. doi: 10.1016/j.bmc.2013.11.025 24393720 10.1016/j.bmc.2013.11.025 PMC4447240

[22] Williams AL, Bohnsack BL. Neural crest derivatives in ocular development: Discerning the eye of the storm. Birth Defects Res Part C Embryo Today Rev. 2015; doi: 10.1002/bdrc.21095 26043871 10.1002/bdrc.21095 PMC5262495

[23] Morgan CA, Parajuli B, Buchman CD, Dria K, Hurley TD. N,N-diethylaminobenzaldehyde (DEAB) as a substrate and mechanism-based inhibitor for human ALDH isoenzymes. Chem Biol Interact. 2015;234: 18–28. doi: 10.1016/j.cbi.2014.12.008 25512087 10.1016/j.cbi.2014.12.008 PMC4414715

[24] Murali D, Yoshikawa S, Corrigan RR, Plas DJ, Crair MC, Oliver G, et al Distinct developmental programs require different levels of Bmp signaling during mouse retinal development. Dev Camb Engl. 2005;132: 913–923. doi: 10.1242/dev.01673 15673568 10.1242/dev.01673

[25] Heermann S, Schütz L, Lemke S, Krieglstein K, Wittbrodt J. Eye morphogenesis driven by epithelial flow into the optic cup facilitated by modulation of bone morphogenetic protein. eLife. 2015;4 doi: 10.7554/eLife.05216 25719386 10.7554/eLife.05216 PMC4337729

[26] Yu PB, Hong CC, Sachidanandan C, Babitt JL, Deng DY, Hoyng SA, et al Dorsomorphin inhibits BMP signals required for embryogenesis and iron metabolism. Nat Chem Biol. 2008;4: 33–41. doi: 10.1038/nchembio.2007.54 18026094 10.1038/nchembio.2007.54 PMC2727650

[27] Hao J, Ho JN, Lewis JA, Karim KA, Daniels RN, Gentry PR, et al In vivo structure-activity relationship study of dorsomorphin analogues identifies selective VEGF and BMP inhibitors. ACS Chem Biol. 2010;5: 245–253. doi: 10.1021/cb9002865 20020776 10.1021/cb9002865 PMC2825290

[28] Zou H, Wieser R, Massagué J, Niswander L. Distinct roles of type I bone morphogenetic protein receptors in the formation and differentiation of cartilage. Genes Dev. 1997;11: 2191–2203. 9303535 10.1101/gad.11.17.2191 PMC275391

[29] Wang S-S, Huang H-Y, Chen S-Z, Li X, Zhang W-T, Tang Q-Q. Gdf6 induces commitment of pluripotent mesenchymal C3H10T1/2 cells to the adipocyte lineage. FEBS J. 2013;280: 2644–2651. doi: 10.1111/febs.12256 23527555 10.1111/febs.12256

[30] den Hollander AI, Biyanwila J, Kovach P, Bardakjian T, Traboulsi EI, Ragge NK, et al Genetic defects of GDF6 in the zebrafish out of sight mutant and in human eye developmental anomalies. BMC Genet. 2010;11: 102 doi: 10.1186/1471-2156-11-102 21070663 10.1186/1471-2156-11-102 PMC2992036

[31] French CR, Stach TR, March LD, Lehmann OJ, Waskiewicz AJ. Apoptotic and proliferative defects characterize ocular development in a microphthalmic BMP model. Invest Ophthalmol Vis Sci. 2013;54: 4636–4647. doi: 10.1167/iovs.13-11674 23737474 10.1167/iovs.13-11674

[32] Zhang XM, Yang XJ. Temporal and spatial effects of Sonic hedgehog signaling in chick eye morphogenesis. Dev Biol. 2001;233: 271–290. doi: 10.1006/dbio.2000.0195 11336495 10.1006/dbio.2000.0195 PMC7048387

[33] Snelson CD, Santhakumar K, Halpern ME, Gamse JT. Tbx2b is required for the development of the parapineal organ. Dev Camb Engl. 2008;135: 1693–1702. doi: 10.1242/dev.016576 18385257 10.1242/dev.016576 PMC2810831

[34] Weiss O, Kaufman R, Mishani E, Inbal A. Ocular vessel patterning in zebrafish is indirectly regulated by Hedgehog signaling. Int J Dev Biol. 2017;61: 277–284. doi: 10.1387/ijdb.160273ai 28621424 10.1387/ijdb.160273ai

[35] Villarroel CE, Villanueva-Mendoza C, Orozco L, Alcántara-Ortigoza MA, Jiménez DF, Ordaz JC, et al Molecular analysis of the PAX6 gene in Mexican patients with congenital aniridia: report of four novel mutations. Mol Vis. 2008;14: 1650–1658. 18776953 PMC2530489

[36] Ramirez-Miranda A, Zenteno JC. PAX6 gene intragenic deletions in Mexican patients with congenital aniridia. Mol Vis. 2006;12: 318–323. 16617299

[37] Jethani J, Sharma VR, Marwah K. Superior Lens Coloboma with Superior Rectus Palsy and Congenital Ptosis. J Optom. 2009;2: 67–69. doi: 10.3921/joptom.2009.67

[38] Mann I, Ross JA. A CASE OF ATYPICAL COLOBOMA ASSOCIATED WITH ABNORMAL RETINAL VESSELS. Br J Ophthalmol. 1929;13: 608–612. 18168834 10.1136/bjo.13.12.608 PMC512133

[39] Abouzeid H, Meire FM, Osman I, ElShakankiri N, Bolay S, Munier FL, et al A new locus for congenital cataract, microcornea, microphthalmia, and atypical iris coloboma maps to chromosome 2. Ophthalmology. 2009;116: 154–162.e1. doi: 10.1016/j.ophtha.2008.08.044 19004499 10.1016/j.ophtha.2008.08.044

[40] Abouzeid H, Boisset G, Favez T, Youssef M, Marzouk I, Shakankiry N, et al Mutations in the SPARC-related modular calcium-binding protein 1 gene, SMOC1, cause waardenburg anophthalmia syndrome. Am J Hum Genet. 2011;88: 92–98. doi: 10.1016/j.ajhg.2010.12.002 21194680 10.1016/j.ajhg.2010.12.002 PMC3014360

[41] Asai-Coakwell M, French CR, Berry KM, Ye M, Koss R, Somerville M, et al GDF6, a novel locus for a spectrum of ocular developmental anomalies. Am J Hum Genet. 2007;80: 306–315. doi: 10.1086/511280 17236135 10.1086/511280 PMC1785352

[42] Bakrania P, Efthymiou M, Klein JC, Salt A, Bunyan DJ, Wyatt A, et al Mutations in BMP4 cause eye, brain, and digit developmental anomalies: overlap between the BMP4 and hedgehog signaling pathways. Am J Hum Genet. 2008;82: 304–319. doi: 10.1016/j.ajhg.2007.09.023 18252212 10.1016/j.ajhg.2007.09.023 PMC2427285

[43] Adler R, Belecky-Adams TL. The role of bone morphogenetic proteins in the differentiation of the ventral optic cup. Dev Camb Engl. 2002;129: 3161–3171. 10.1242/dev.129.13.3161 12070091

[44] Asai-Coakwell M, March L, Dai XH, Duval M, Lopez I, French CR, et al Contribution of growth differentiation factor 6-dependent cell survival to early-onset retinal dystrophies. Hum Mol Genet. 2013;22: 1432–1442. doi: 10.1093/hmg/dds560 23307924 10.1093/hmg/dds560

[45] Weston CR, Wong A, Hall JP, Goad MEP, Flavell RA, Davis RJ. JNK initiates a cytokine cascade that causes Pax2 expression and closure of the optic fissure. Genes Dev. 2003;17: 1271–1280. doi: 10.1101/gad.1087303 12756228 10.1101/gad.1087303 PMC196061

[46] Bielen H, Houart C. BMP signaling protects telencephalic fate by repressing eye identity and its Cxcr4-dependent morphogenesis. Dev Cell. 2012;23: 812–822. doi: 10.1016/j.devcel.2012.09.006 23079599 10.1016/j.devcel.2012.09.006 PMC7116079

[47] Pant SD, March LD, Famulski JK, French CR, Lehmann OJ, Waskiewicz AJ. Molecular mechanisms regulating ocular apoptosis in zebrafish gdf6a mutants. Invest Ophthalmol Vis Sci. 2013;54: 5871–5879. doi: 10.1167/iovs.12-11315 23847306 10.1167/iovs.12-11315

[48] Abouzeid H, Favez T, Schmid A, Agosti C, Youssef M, Marzouk I, et al Mutations in ALDH1A3 represent a frequent cause of microphthalmia/anophthalmia in consanguineous families. Hum Mutat. 2014;35: 949–953. doi: 10.1002/humu.22580 24777706 10.1002/humu.22580

[49] Veien ES, Rosenthal JS, Kruse-Bend RC, Chien C-B, Dorsky RI. Canonical Wnt signaling is required for the maintenance of dorsal retinal identity. Dev Camb Engl. 2008;135: 4101–4111. doi: 10.1242/dev.027367 19004855 10.1242/dev.027367 PMC2667153

[50] Liu C, Widen SA, Williamson KA, Ratnapriya R, Gerth-Kahlert C, Rainger J, et al A secreted WNT-ligand-binding domain of FZD5 generated by a frameshift mutation causes autosomal dominant coloboma. Hum Mol Genet. 2016;25: 1382–1391. doi: 10.1093/hmg/ddw020 26908622 10.1093/hmg/ddw020 PMC4787907

[51] Henske EP, Jóźwiak S, Kingswood JC, Sampson JR, Thiele EA. Tuberous sclerosis complex. Nat Rev Dis Primer. 2016;2: 16035 doi: 10.1038/nrdp.2016.35 27226234 10.1038/nrdp.2016.35

[52] Nakamura KM, Diehl NN, Mohney BG. Incidence, ocular findings, and systemic associations of ocular coloboma: a population-based study. Arch Ophthalmol Chic Ill 1960. 2011;129: 69–74. doi: 10.1001/archophthalmol.2010.320 21220631 10.1001/archophthalmol.2010.320 PMC3126628

[53] Fitzgerald DE, Chung I, Krumholtz I. An analysis of high myopia in a pediatric population less than 10 years of age. Optom St Louis Mo. 2005;76: 102–114. 10.1016/s1529-1839(05)70263-3 15732627

[54] Barrett BT, Bradley A, Candy TR. The relationship between anisometropia and amblyopia. Prog Retin Eye Res. 2013;36: 120–158. doi: 10.1016/j.preteyeres.2013.05.001 23773832 10.1016/j.preteyeres.2013.05.001 PMC3773531

[55] Hartsock A, Lee C, Arnold V, Gross JM. In vivo analysis of hyaloid vasculature morphogenesis in zebrafish: A role for the lens in maturation and maintenance of the hyaloid. Dev Biol. 2014;394: 327–339. doi: 10.1016/j.ydbio.2014.07.024 25127995 10.1016/j.ydbio.2014.07.024 PMC4172555

[56] Weiss O, Kaufman R, Michaeli N, Inbal A. Abnormal vasculature interferes with optic fissure closure in lmo2 mutant zebrafish embryos. Dev Biol. 2012;369: 191–198. doi: 10.1016/j.ydbio.2012.06.029 22819672 10.1016/j.ydbio.2012.06.029

[57] Siemerink MJ, Augustin AJ, Schlingemann RO. Mechanisms of ocular angiogenesis and its molecular mediators. Dev Ophthalmol. 2010;46: 4–20. doi: 10.1159/000320006 20703029 10.1159/000320006

[58] Beaulieu CL, Majewski J, Schwartzentruber J, Samuels ME, Fernandez BA, Bernier FP, et al FORGE Canada Consortium: outcomes of a 2-year national rare-disease gene-discovery project. Am J Hum Genet. 2014;94: 809–817. doi: 10.1016/j.ajhg.2014.05.003 24906018 10.1016/j.ajhg.2014.05.003 PMC4121481

[59] Rembold M, Loosli F, Adams RJ, Wittbrodt J. Individual cell migration serves as the driving force for optic vesicle evagination. Science. 2006;313: 1130–1134. doi: 10.1126/science.1127144 16931763 10.1126/science.1127144

[60] Proulx K, Lu A, Sumanas S. Cranial vasculature in zebrafish forms by angioblast cluster-derived angiogenesis. Dev Biol. 2010;348: 34–46. doi: 10.1016/j.ydbio.2010.08.036 20832394 10.1016/j.ydbio.2010.08.036

[61] Choi J, Dong L, Ahn J, Dao D, Hammerschmidt M, Chen J-N. FoxH1 negatively modulates flk1 gene expression and vascular formation in zebrafish. Dev Biol. 2007;304: 735–744. doi: 10.1016/j.ydbio.2007.01.023 17306248 10.1016/j.ydbio.2007.01.023 PMC1876740

[62] Hamburger V, Hamilton HL. A series of normal stages in the development of the chick embryo. J Morphol. 1951;88: 49–92. doi: 10.1002/jmor.1050880104 24539719

[63] Eom DS, Amarnath S, Fogel JL, Agarwala S. Bone morphogenetic proteins regulate neural tube closure by interacting with the apicobasal polarity pathway. Dev Camb Engl. 2011;138: 3179–3188. doi: 10.1242/dev.058602 21750029 10.1242/dev.058602 PMC3133910

[64] Amarnath S, Agarwala S. Cell-cycle-dependent TGFβ-BMP antagonism regulates neural tube closure by modulating tight junctions. J Cell Sci. 2017;130: 119–131. doi: 10.1242/jcs.179192 27034139 10.1242/jcs.179192 PMC5394770

[65] Halfter W, Von Boxberg Y. Axonal Growth on Solubilized and Reconstituted Matrix from the Embryonic Chicken Retina Inner Limiting Membrane. Eur J Neurosci. 1992;4: 840–852. 12106307 10.1111/j.1460-9568.1992.tb00194.x

[66] Cermak T, Doyle EL, Christian M, Wang L, Zhang Y, Schmidt C, et al Efficient design and assembly of custom TALEN and other TAL effector-based constructs for DNA targeting. Nucleic Acids Res. 2011;39: e82 doi: 10.1093/nar/gkr218 21493687 10.1093/nar/gkr218 PMC3130291

[67] Pillay LM, Forrester AM, Erickson T, Berman JN, Waskiewicz AJ. The Hox cofactors Meis1 and Pbx act upstream of gata1 to regulate primitive hematopoiesis. Dev Biol. 2010;340: 306–317. doi: 10.1016/j.ydbio.2010.01.033 20123093 10.1016/j.ydbio.2010.01.033

