# Supplementary material for: Morphogenetic defects underlie Superior Coloboma, a newly identified closure disorder of the dorsal eye
Source: PLoS Genet. 2018 Mar 9;14(3):e1007246. doi: 10.1371/journal.pgen.1007246 (PMC5862500; doi:10.1371/journal.pgen.1007246)
Supplement: S1 Table — (DOCX) [file pgen.1007246.s001.docx]

| **Patient #** | **Age at diagnosis** | **Laterality and eye(s) affected** | **Ocular Phenotype** | **Other findings** | **Clinical Center** |
| --- | --- | --- | --- | --- | --- |
| #1 | 23 years | Unilateral, OS | Iris coloboma, with anomalous retinal vasculature and localized sheathing of retinal arteries | Tuberous Sclerosis | Edmonton, Canada |
| #2 | 21 years | Bilateral | Asymmetric phenotypic severity:  OD. Two small superior iris lesions, with pupillary distortion  OS. Large superior iris coloboma, small superior lenticular (lens) coloboma with a small defect in the lens zonule | Congenital glaucoma,  Parental consanguinity | Edmonton, Canada |
| #3 | 8 months | Unilateral, OS | Unilateral lenticular coloboma | Nil | Edmonton, Canada |
| #4 | 14 months | Unilateral, OD | Superior scleral defect with superior retinal colobomatous changes, Situs inversus (displaced vessels) | Nil | Edmonton, Canada |
| #5 | 23 years | Unilateral, OS | Unilateral superior retinal and optic nerve coloboma | Dandy-Walker syndrome | Edmonton, Canada |
| #6 | 5 years | Unilateral, OD | Iris and retinochoroidal coloboma | Tri-atrial heart | National Eye Institute, USA |
| #7 | 2 years | Unilateral, OD | Retinochoroidal coloboma and mild microphthalmia | Ovarian torsion in infancy | Cambridge, UK |
| #8 | 2 months | Unilateral, OS | Iris coloboma with microphthalmia. Right eye is microphthalmic with complete corneal opacity. | Cardiac and renal anomalies. Epilepsy | University of Michigan, USA |

OD=right eye, OS=left eye
