# Supplementary figures and images for: Morphogenetic defects underlie Superior Coloboma, a newly identified closure disorder of the dorsal eye
Source: PLoS Genet. 2018 Mar 9;14(3):e1007246. doi: 10.1371/journal.pgen.1007246 (PMC5862500; doi:10.1371/journal.pgen.1007246)

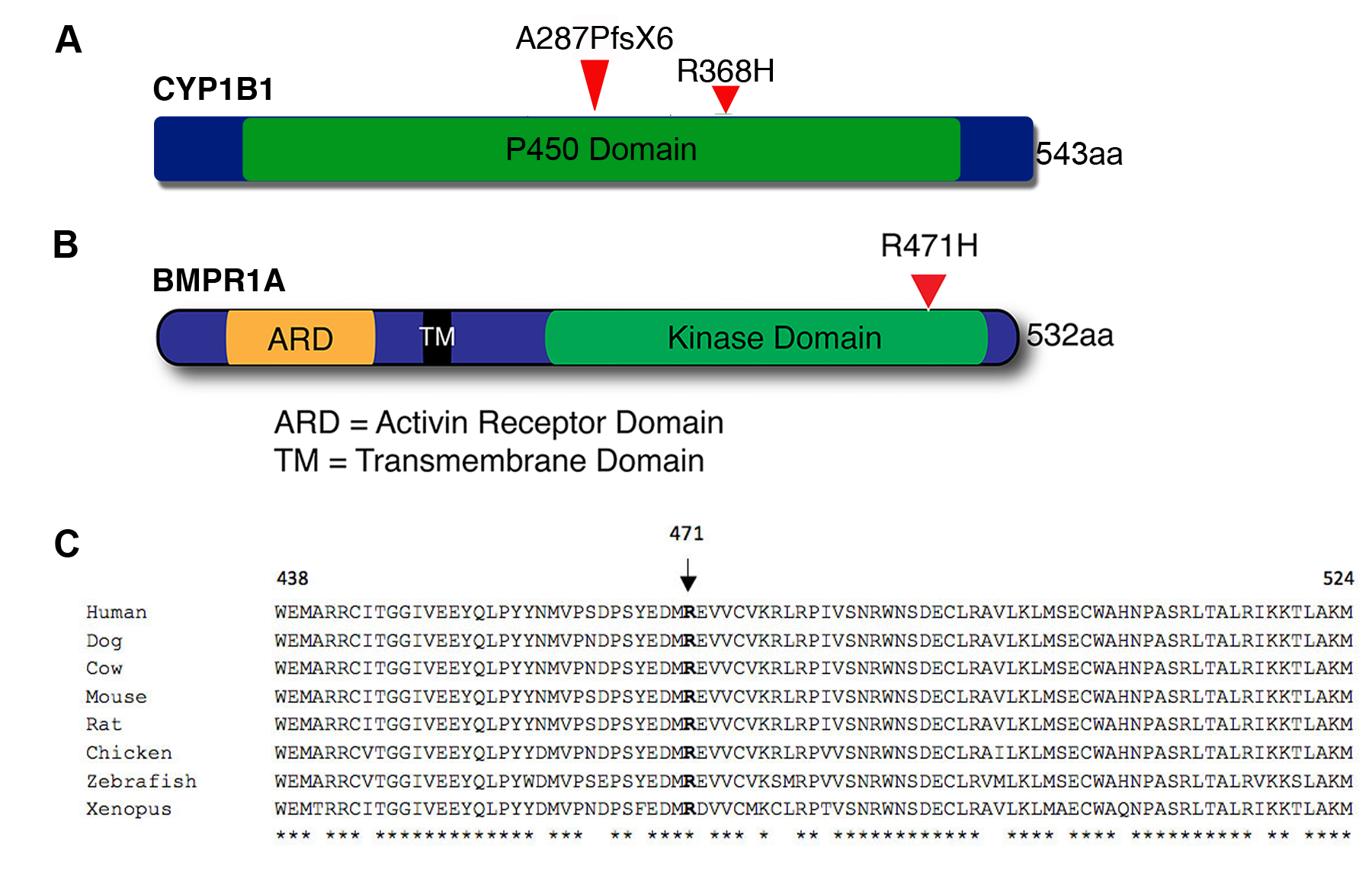

Supplement: S1 Fig — (A) Diagram of the human CYP1B1 protein, with the compound heterozygous mutations carried by patient#2 indicated. (B) Diagram of the human BMPR1A protein showing rare variant present in patient#2. (C) Alignment illustrating the evolutionary conservation of the BMPR1A protein kinase domain. The altered residue (p.R471H) is depicted in bold, with invariant residues denoted by *. (TIF) [file pgen.1007246.s003.tif]

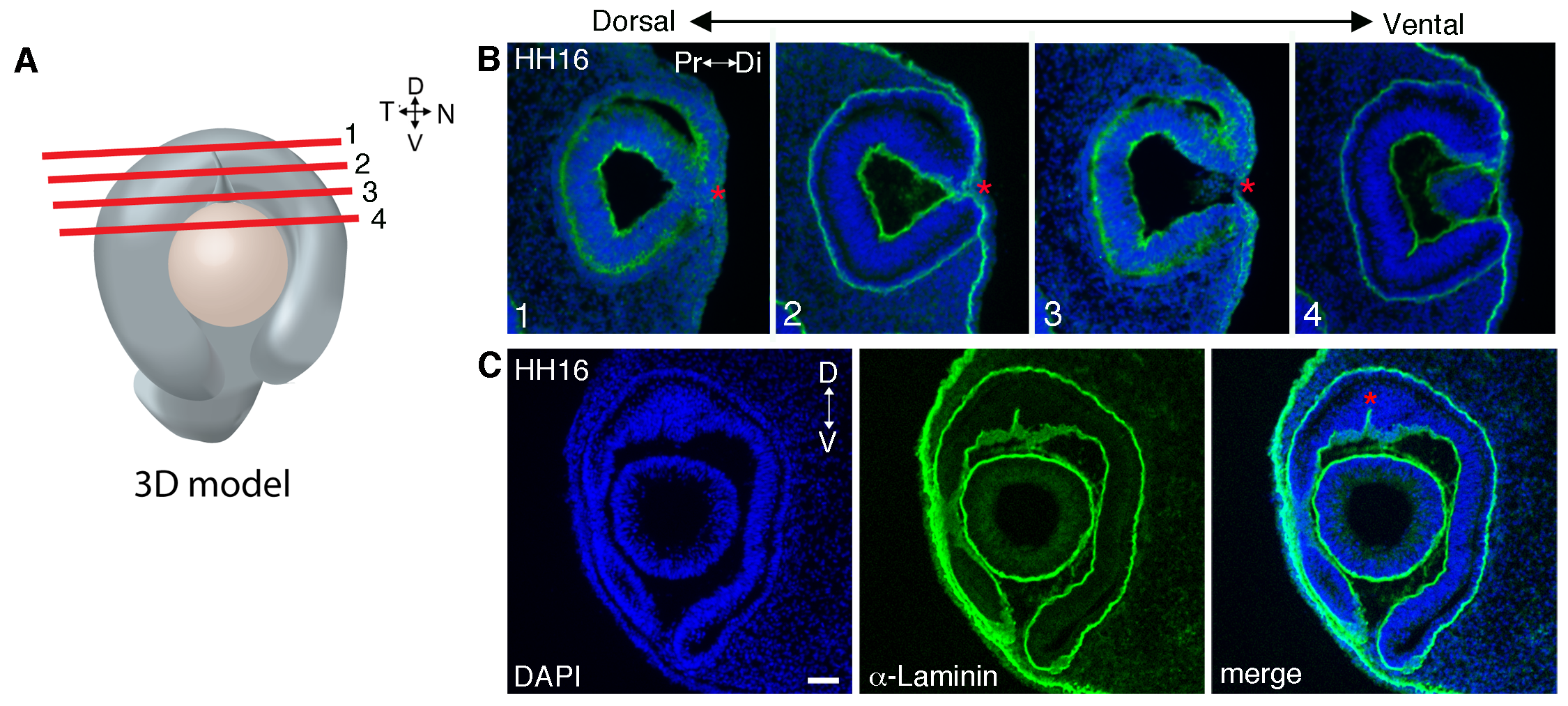

Supplement: S2 Fig — (A) 3D Model of the eye depicting where the eye was sectioned to create the serial horizontal sections shown in B. B) Serial cryostat sections of a chick HH16 stage eye stained with DAPI (blue) and α-Laminin antibody (green). First three sections are dorsal to the lens and third one is through the lens. (C) Tangential section of HH16 chick eye labeled with DAPI (blue) and α-Laminin antibody (green). D, dorsal; V, ventral; Di, distal; Pr, proximal; HH, Hamburger Hamilton. Red asterisks indicate superior ocular sulcus. Scale bar is 50 μm. (TIF) [file pgen.1007246.s004.tif]

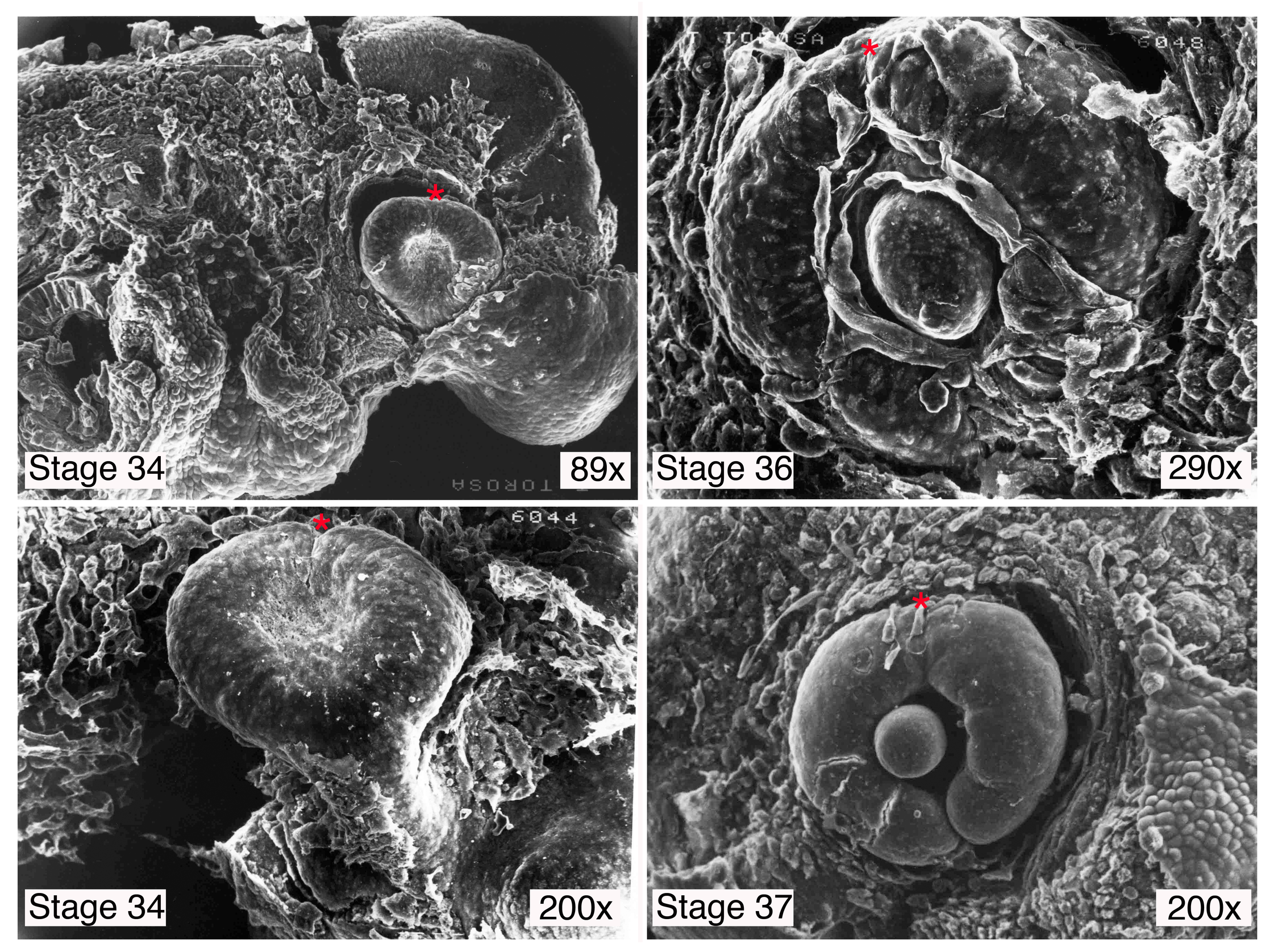

Supplement: S3 Fig — Scanning electron microscopy images of newt (Taricha tarosa) ocular development. Panels on left display SEM images of stage 34 embryos after partial dissection of surface tissues. Panels on right show slightly older embryos (stage 36–37), with vasculature intact in the stage 36 example. Red asterisks indicate superior ocular sulcus. (TIF) [file pgen.1007246.s005.tif]

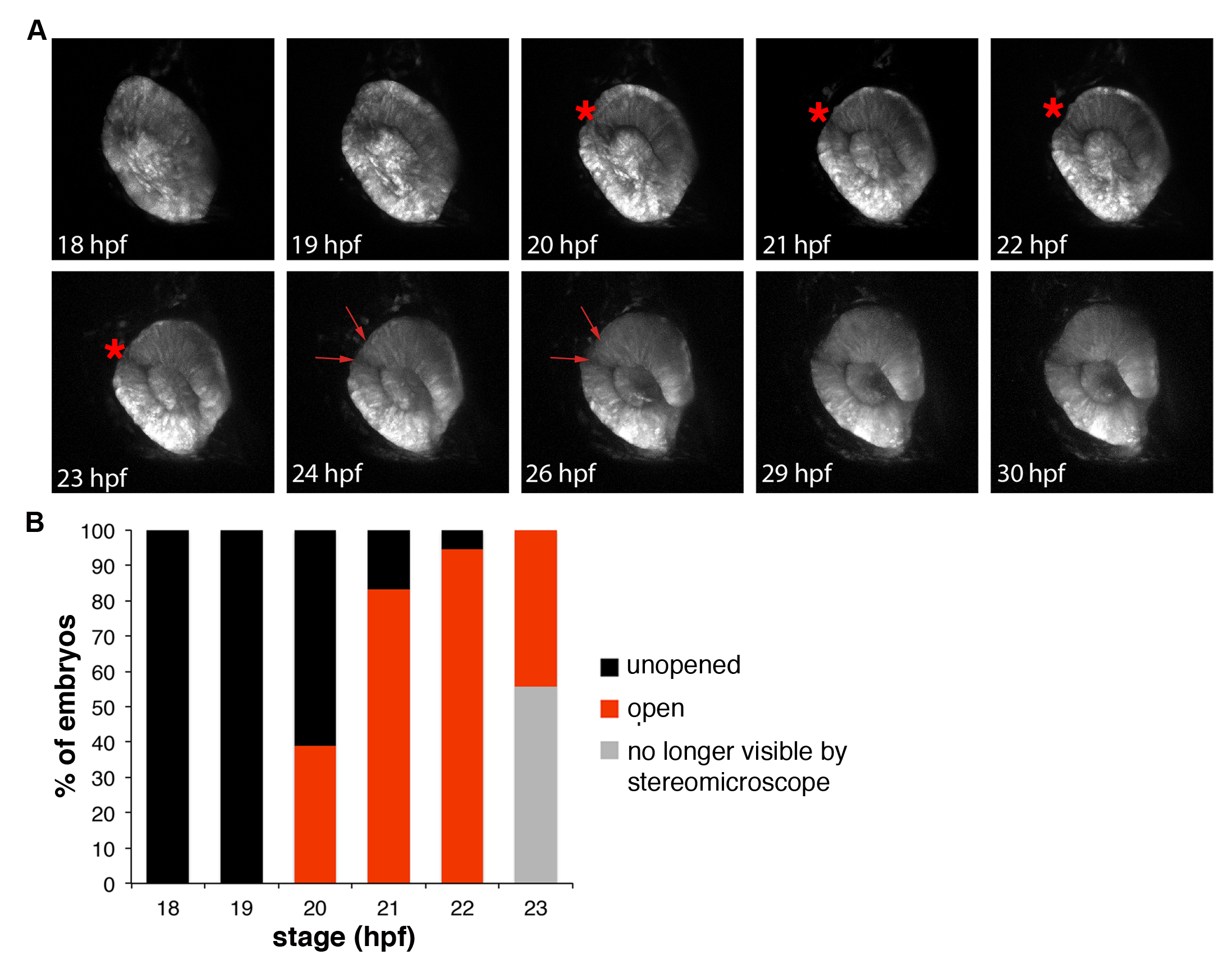

Supplement: S4 Fig — (A) Time-lapse images showing lateral views of the eye of a Tg(rx3:GFP) embryo. The superior ocular sulcus appears as a narrow groove across the dorsal retina at ~20 hpf (red asterisk), becomes wider by 24 hpf (red arrows) and disappears after 26 hpf. (B) Timing of SOS as viewed under a stereomicroscope. The wide and shallow phase is not visible by stereomicroscope, so the red bars indicate the percentage of embryos with a narrow and distinct sulcus. (TIF) [file pgen.1007246.s006.tif]

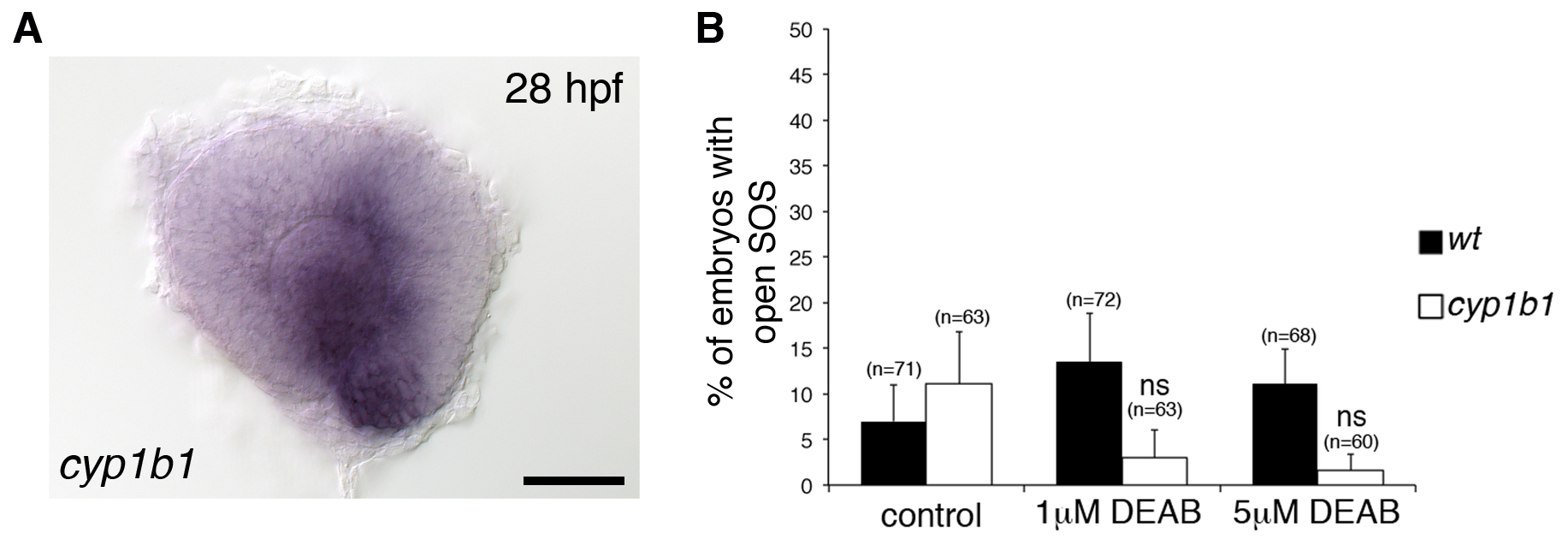

Supplement: S5 Fig — (A) Lateral view of a 28 hpf zebrafish eye following in situ hybridization for cyp1b1. Note that expression extends into the dorsal eye. (B) Quantification of open SOS in 28 hpf embryos from cyp1b1+/- incrosses treated from 10 hpf with control solution or the Aldh inhibitor DEAB. N = 3 experiments, n = number of embryos. Data are means ± SEM. Statistics is two-way ANOVA with Tukey's test. Scale bar is 50 μm. ns, not significant. (TIF) [file pgen.1007246.s007.tif]

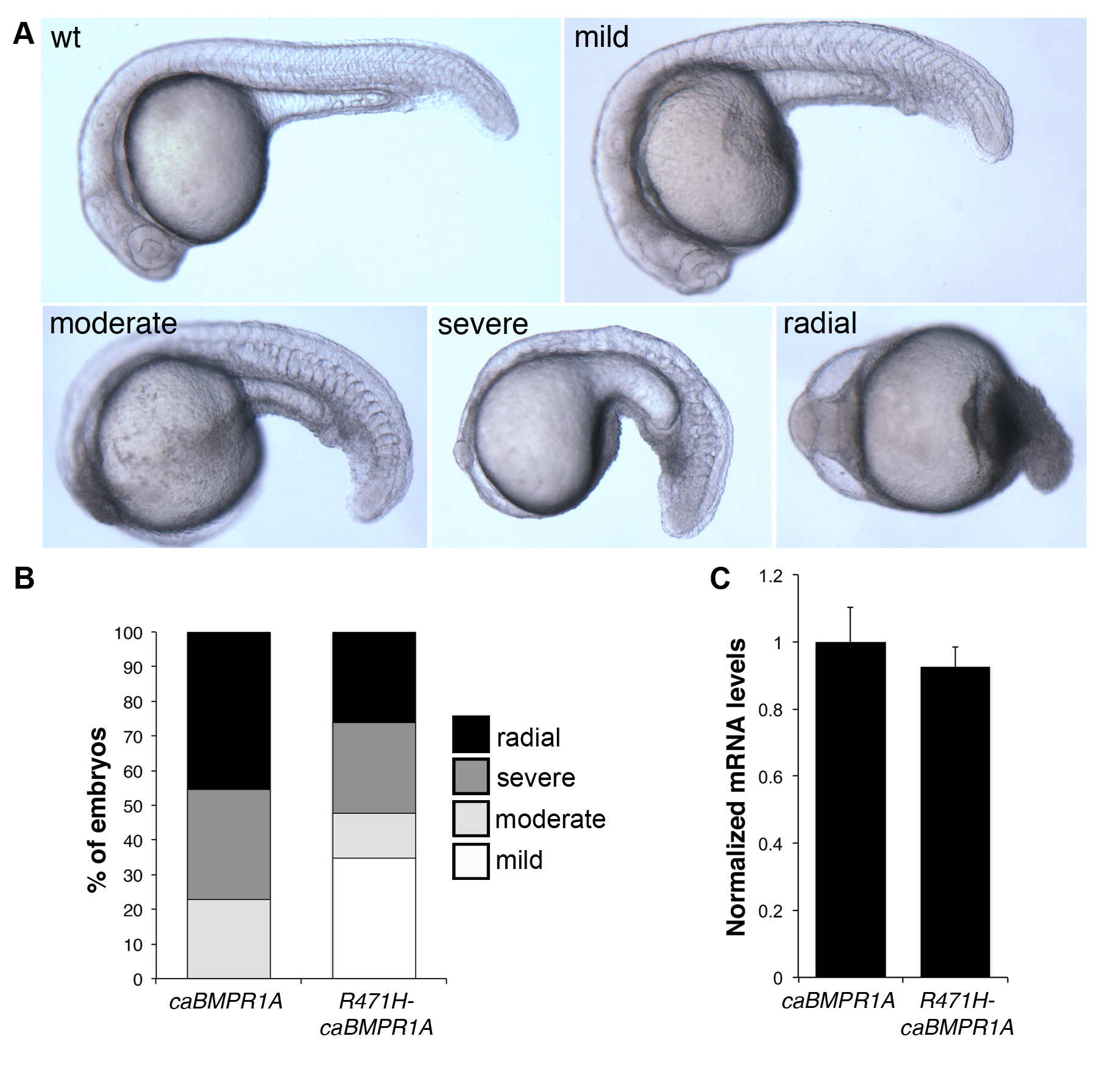

Supplement: S6 Fig — (A) One cell-stage zebrafish embryos were injected with caBMPR1A or R471H-caBMPR1A mRNA, and assessed at 24 hpf for morphological abnormalities by categorization according to the pictures shown. (B) Graph showing percentage of embryos injected with caBMPR1A (n = 22 embryos) or R471H-caBMPR1A (n = 23 embryos) that fit into each category of morphological abnormality. (C) qPCR showing equal amounts of injected RNA for each condition. Statistics is two-tailed t test. (TIF) [file pgen.1007246.s008.tif]

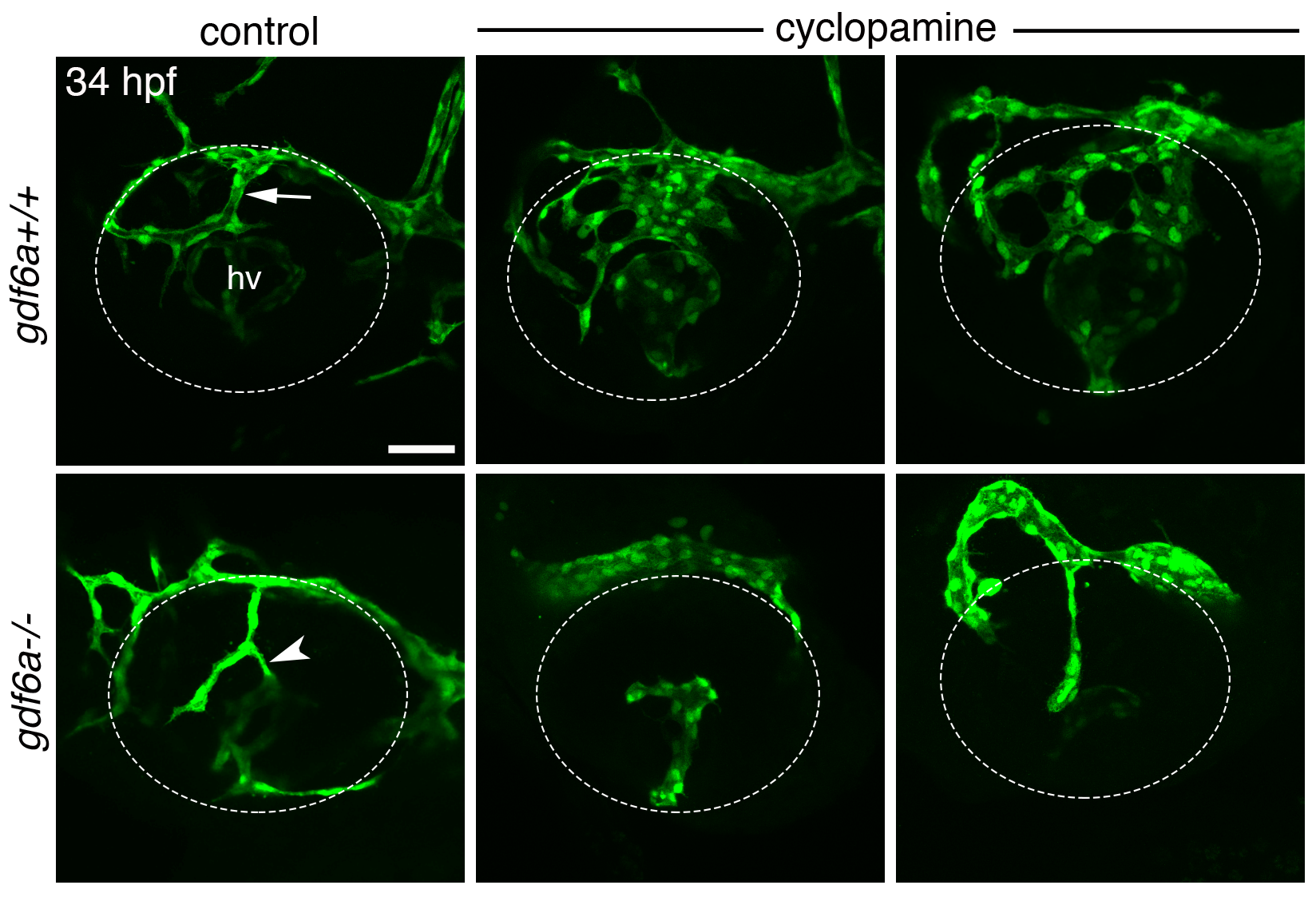

Supplement: S7 Fig — Maximum projection confocal images of 34 hpf eyes from Tg(kdrl:eGFP);gdf6a+/+ and Tg(kdrl:eGFP);gdf6a-/- zebrafish embryos following treatment with control solution or 10 μM cyclopamine from 10 hpf. Blood vessels fluoresce green and the eye is outlined by dotted lines. DRV is indicated by arrow. Ectopic connection between superficial and hyaloid vasculatures indicated by arrowhead. Top row, right two panels are two examples of vessel overgrowth phenotype in cyclopamine-treated wildtype embryos. Bottom row, middle and right panels show the eyes of cyclopamine-treated gdf6a-/- embryos that either failed to form a DRV (n = 3/6 embryos) or grew a simple DRV that did not make an ectopic connection to the hyaloid vasculature (n = 3/6 embryos), respectively. hv, hyaloid vasculature. Scale bar is 50 μm. (TIF) [file pgen.1007246.s009.tif]

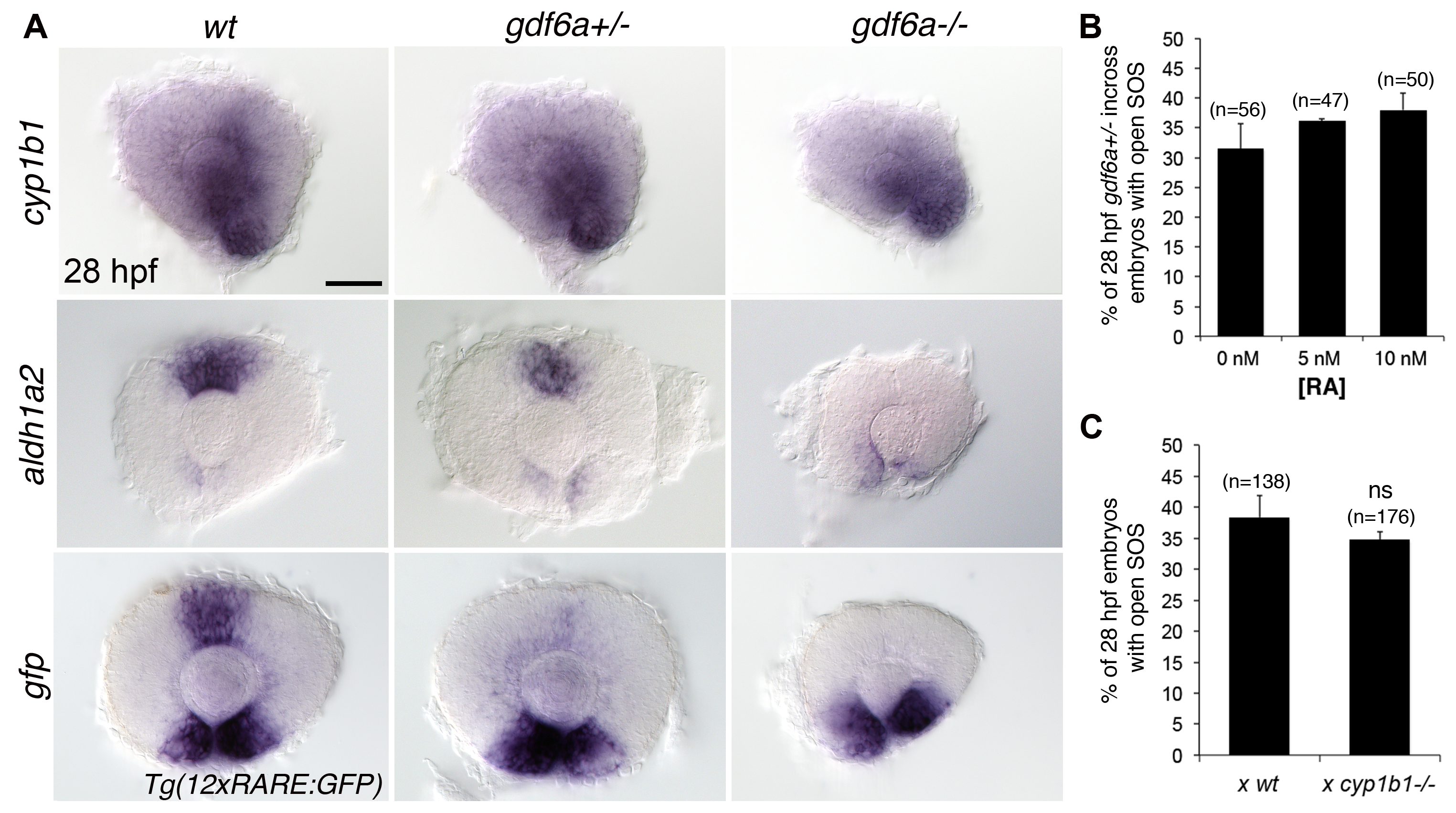

Supplement: S8 Fig — (A) Lateral views of eyes from 28 hpf zebrafish embryos that are gdf6a+/+, gdf6a +/-, or gdf6a-/- and have been processed for in situ hybridization. The top two rows show expression of the retinoic acid-synthesis genes cyp1b1 and aldha1a2. The bottom row shows expression of GFP in transgenic zebrafish carrying a reporter for RA signaling [Tg(12xRARE:GFP)] and are also gdf6a+/+, gdf6a +/-, or gdf6a-/-. Note reduced RA signaling in the superior retina of gdf6a +/- and gdf6a-/- embryos. (B) Graph showing no effect of retinoic acid treatment on SOS closure. Embryos from gdf6a+/- incrosses were grown from 10 hpf in control media, 5 nM retinoic acid, or 10 nM retinoic acid, and assessed at 28 hpf for an open SOS. (C) Graph showing no effect of the cyp1b1 mutation on SOS closure in gdf6a heterozygotes. gdf6a+/-;cyp1b+/- fish were crossed to wildtype or cyp1b-/- fish and the percentage of embryos with an open SOS was assessed at 28 hpf. n = number of embryos, N = 2 (B) or 3 experiments (C). Data are means ± SEM. ns, not significant (TIF) [file pgen.1007246.s010.tif]
